# Serum Metabolic Fingerprints on Bowl-Shaped Submicroreactor Chip for Chemotherapy Monitoring

**DOI:** 10.1021/acsnano.1c09864

**Published:** 2022-01-31

**Authors:** Xia Yin, Jing Yang, Mengji Zhang, Xinyao Wang, Wei Xu, Cameron-Alexander H. Price, Lin Huang, Wanshan Liu, Haiyang Su, Wenjing Wang, Hongyu Chen, Guangjin Hou, Mark Walker, Ying Zhou, Zhen Shen, Jian Liu, Kun Qian, Wen Di

**Affiliations:** † State Key Laboratory for Oncogenes and Related Genes, Shanghai Key Laboratory of Gynecologic Oncology, Department of Obstetrics and Gynecology, Renji Hospital, School of Medicine, Shanghai Jiao Tong University, Shanghai, 200127, P.R. China; ‡ School of Biomedical Engineering and Med-X Research Institute, Shanghai Jiao Tong University, Shanghai, 200030, P.R. China; ○ State Key Laboratory of Catalysis, Dalian Institute of Chemical Physics, Chinese Academy of Sciences, Dalian, Liaoning 116023, P.R. China; ¶ The University of Manchester at Harwell, Diamond Light Source, Didcot, Oxfordshire OX11 0DE, U.K.; § UK Catalysis Hub, Research Complex at Harwell, Rutherford Appleton Laboratories, Harwell Campus, Didcot, Oxfordshire OX11 0FA, U.K.; ⊥ Department of Obstetrics and Gynecology, University of Ottawa, Ottawa, Ontario ON K1H 8L6, Canada; ∥ Department of Obstetrics and Gynecology, The First Affiliated Hospital of USTC, Division of Life Sciences and Medicine, University of Science and Technology of China, Hefei, Auhui 230001, P.R. China; ∇ DICP-Surrey Joint Centre for Future Materials, Department of Chemical and Process Engineering, and Advanced Technology Institute, University of Surrey, Guilford, Surrey GU2 7XH, U.K.

**Keywords:** submicroreactor, anisotropic particles, metabolites, chemotherapy, mass spectrometry

## Abstract

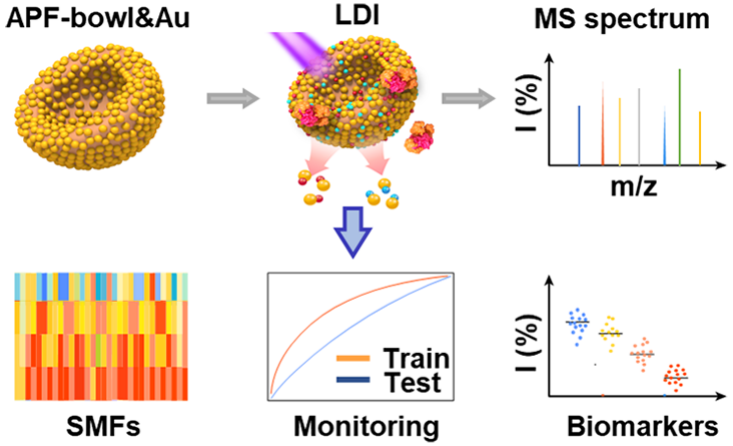

Chemotherapy is a primary cancer treatment strategy, the monitoring of which is critical to enhancing the survival rate and quality of life of cancer patients. However, current chemotherapy monitoring mainly relies on imaging tools with inefficient sensitivity and radiation invasiveness. Herein, we develop the bowl-shaped submicroreactor chip of Au-loaded 3-aminophenol formaldehyde resin (denoted as APF-bowl&Au) with a specifically designed structure and Au loading content. The obtained APF-bowl&Au, used as the matrix of laser desorption/ionization mass spectrometry (LDI MS), possesses an enhanced localized electromagnetic field for strengthened small metabolite detection. The APF-bowl&Au enables the extraction of serum metabolic fingerprints (SMFs), and machine learning of the SMFs achieves chemotherapy monitoring of ovarian cancer with area-under-the-curve (AUC) of 0.81–0.98. Furthermore, a serum metabolic biomarker panel is preliminarily identified, exhibiting gradual changes as the chemotherapy cycles proceed. This work provides insights into the development of nanochips and contributes to a universal detection platform for chemotherapy monitoring.

Chemotherapy is one of the leading treatment strategies applied in over 30% of cancer patients.^1^ In particular, it plays an indispensable role in treating metastatic cancer, which cannot be directly eradicated by surgery or radiotherapy.^2−4^ Importantly, chemotherapy monitoring is essential to avoid complications, minimize side effects, and reduce medical costs, increasing cancer patients’ survival rate and quality of life. To date, chemotherapy monitoring primarily relies on imaging methods, with the intrinsic drawbacks of suboptimal sensitivity, radiation invasiveness, and restriction to solid tumors only.^5,6^ In addition, large imaging instruments hinder the potential use in point-of-care testing (POCT) and regions with limited resources. In contrast, high-performance blood tests of biomarkers may offer corresponding merits of high sensitivity, fast analytical speed, low invasiveness, and general adaptability toward POCT and universal use.^7,8^ Furthermore, small metabolites^9^ in the serum provide a more real-time physiological state of the human body compared to the nucleic acids and proteins, displaying better feasibility for monitoring use.^8,10,11^ Therefore, a high-performance detection platform is in urgent demand to profile the serum metabolic fingerprints (SMFs) for characterizing the pathological process of disease (*e.g.*, chemotherapy monitoring).

Mass spectrometry (MS),^12^ as a primary tool for metabolic analysis, is capable of superior sensitivity and improved molecular identification by the high-resolution recording of metabolites (±10 mDa) in comparison to nuclear magnetic resonance (NMR).^13,14^ Notably, laser desorption ionization (LDI) MS further enhances the detection sensitivity to the femtomolar level, where the matrix plays a critical role in facilitating the solid-to-gas transition.^15,16^ The traditional organic matrix exhibited huge success in macromolecule detection (*e.g.*, protein) as honored by the Nobel Prize;^17,18^ however, its potential use in small metabolite detection has been hindered because of the unwanted fragmentation in the low mass range (<1000 Da) and uneven cocrystallization with biosamples (*e.g.*, serum). Although the exploration of inorganic matrices revealed some promising discoveries to address the current obstacles of the organic matrix, it is far from ideal to detect the small metabolite from complex biosamples. The rational design of the inorganic matrix is key to improve the LDI efficiency toward constructing the next-generation high-performance detection platform.

Noble metal-decorated nanostructures have been widely applied in energy storage, catalysis, and biomedicine,^19−21^ the performance of which is determined by their corresponding micro/nanostructure and composition. Recently, particles with anisotropic architectures (*e.g.*, Janus particles) have attracted more attention over isotropic particles by presenting intriguing properties as matrices in LDI MS,^22,23^ while few of them were utilized for metabolic fingerprint analysis toward biomedical applications caused by complex preparation procedures. The bowl-shaped particle can be fabricated *via* a facile solvent-assisted repolymerization process, and its concave domain enables the trapping of light with long scattering length, which can be desirable for matrix use but has not been explored yet.^24−27^ As for composition, the noble metal nanoparticles present superior light absorption and electrical conductivity that allow for the facilitation of the desorption/ionization of analytes under laser irradiation.^28−30^ In particular, Au nanoparticles display preferable stability and high biocompatibility, serving as a primary candidate for biosample detection.^31,32^ Accordingly, bowl-structured particles decorated with Au nanoparticles as the matrix of LDI MS analysis would allow for strengthened metabolic detection and further conquer the dilemma of current chemotherapy monitoring.

Herein, we report the submicroreactor APF-bowl&Au chip-assisted LDI MS for chemotherapy monitoring of ovarian cancer (Scheme 1). We report various chips of APF&*M*Au (where APF represents 3-aminophenol formaldehyde resin, “&” represents loading, and *M* (=0.72/0.96/1.20) is calculated as the theoretical mass ratio of Au over APF) with an adjustable structure and tunable Au loading content, both of which are rationally designed and synthesized. The desirable properties of laser trapping and charge transfer of the optimized APF-bowl&0.96Au enable the SMFs’ extraction by using only 1 μL of serum per sample within 1 min (Scheme 1a). The chemotherapy cycles of ovarian cancer can be monitored by machine learning of SMFs with area-under-the-curve (AUC) of 0.81–0.98 (Scheme 1b). This work promotes the development of anisotropic submicroreactor chips in metabolic analysis and provides a shortcut for precision prognostics in cancer therapy.

**Scheme 1:**
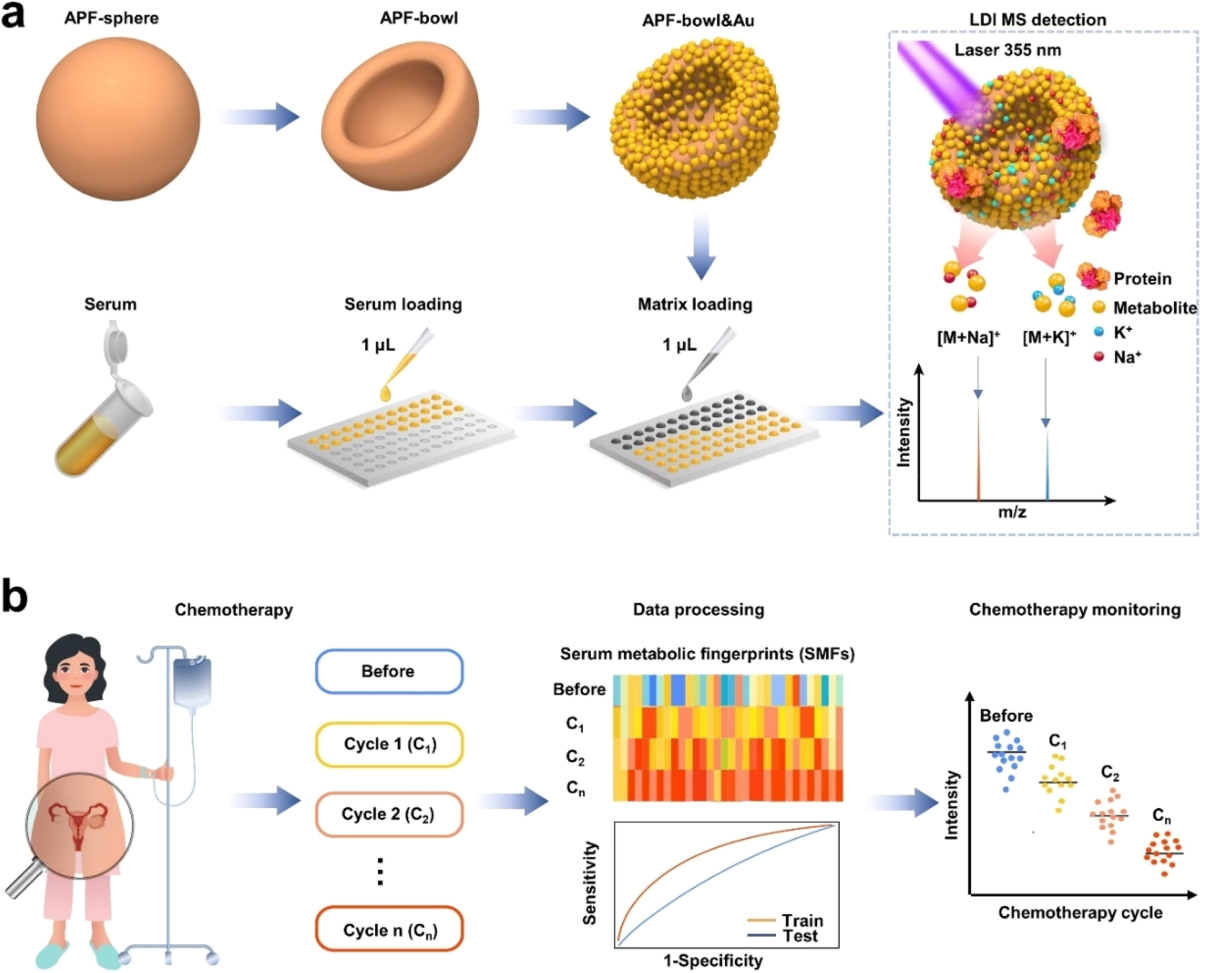
SMFs Extraction by APF-bowl&Au Chip and Chemotherapy Monitoring by Machine Learning Methods (a) The workflow for SMFs acquisition by APF-bowl&Au assisted LDI MS, including the incubation of 1 μL of serum sample with matrix suspension of APF-bowl&Au and detection by LDI MS equipped with Nd:YAG laser (355 nm) for obtaining the cation adducted signals of metabolites ([M + Na]^+^ and [M + K]^+^). The APF-bowl&Au was prepared by etching the APF-sphere with acetone and then introducing the Au nanoparticles. (b) Machine learning of SMFs for chemotherapy monitoring. The SMFs of patients before chemotherapy and after different cycles of chemotherapy, including cycle 1 (C_1_), cycle 2 (C_2_), and cycle *n* (*n* ≥ 5, C_*n*_), could be extracted by data preprocessing of the original mass spectra. The database of SMFs, assisted with machine learning methods, could achieve the chemotherapy monitoring and yielded a preliminarily identified metabolic biomarker panel.

## Results and Discussion

### Morphological and Structural Characterization of APF&Au Chips

**Figure 1:**
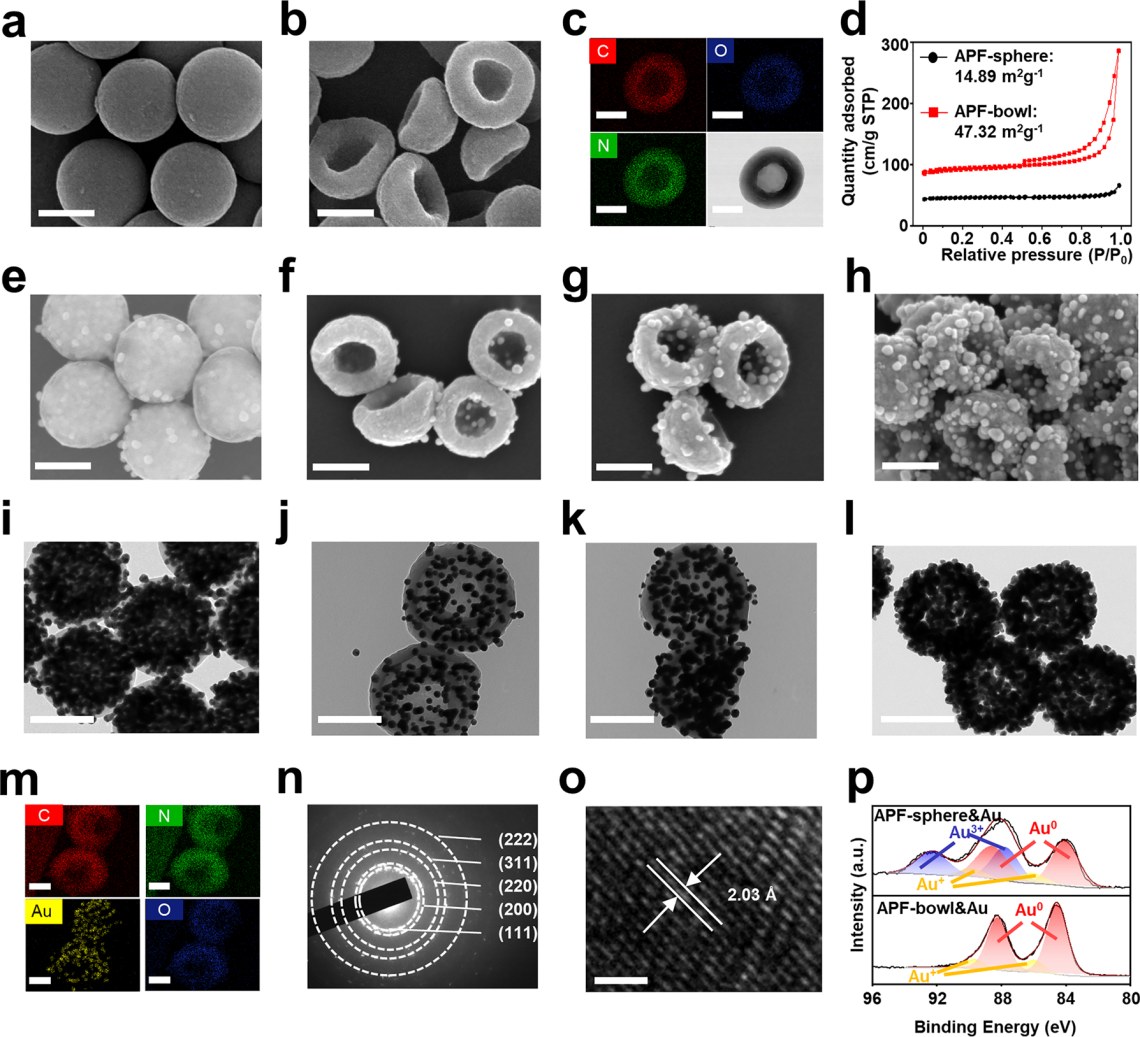
Morphological and structural characterization of APF&Au chips. The SEM images of (a) APF-sphere and (b) APF-bowl. (c) The elemental mapping of APF-bowl containing C (red), O (blue), and N (green). (d) The N_2_ adsorption–desorption isotherms of APF-bowl (red) and APF-sphere (black) with the specific surface area labeled. The SEM images of (e) APF-sphere&Au, (f) APF-bowl&0.72Au, (g) APF-bowl&0.96Au, and (h) APF-bowl&1.20Au. The TEM images of (i) APF-sphere&Au, (j) APF-bowl&0.72Au, (k) APF-bowl&0.96Au, and (l) APF-bowl&1.20Au. (m) The elemental mapping of APF-bowl&Au containing C (red), N (green), Au (yellow), and O (blue). (n) The SAED pattern of APF-bowl&Au with typical rings of (111), (200), (220), (311), and (222) of Au. (o) The HR TEM image displayed the Au crystal lattice of 2.03 Å for (200). (p) The XPS characterizations of APF-sphere&Au (top) and APF-bowl&Au (down). The scale bar is 200 nm in panels a, b, and e–l, 100 nm in panels c and m, and 1 nm in panel o.

The APF&Au chips, prepared according to the procedures in Scheme 1a, were characterized in terms of their morphology and structure (Figure 1). The APF-sphere with elements uniformly distributed (including C, O, and N; see scanning electron microscopy (SEM) image and elemental mapping in Figure 1a and Supporting Figure 1, respectively) were synthesized by the polymerization of 3-aminophenol and formaldehyde for 30 min, showing an average particle size of *ca*. 320 nm. The polymerization and nucleation rate of the APF-sphere is fast at room temperature (<1 min) owing to well-established ongoing phenol-formaldehyde type reaction and nucleophilic addition of amino and aldehydes. Further increase in polymerization degree will mainly occur in the outer layer of the APF-sphere because its intrinsic morphology blocks the contact between the oligomer in the solution and the inner surface. Therefore, the short polymerization process leads to an exploitable inhomogeneity in the inner structure and outer shell composition.^33,34^ Acetone, because it is a polar solvent, can dissolve the inner low polymerization degree APF resin. Similar to a deflation process, the internally dissolved oligomer was driven to flow out under osmotic pressure, and the obtained hollow shells were extruded into bowl-like particles.^24^ The transformation of the sphere into a bowl structure was accomplished rapidly (<5 min) after adding acetone, and the structure was found to be stable (Supporting Figure 2). Because of this, the uniform APF-bowl is similar in size to the original APF-sphere (SEM image shown in Figure 1b and elemental mapping shown in Figure 1c). Notably, APF-bowls with their specific open-structure possessed a specific surface area of 47.32 m^2^ g^–1^, which is much larger than that of APF-sphere of 14.89 m^2^ g^–1^, as revealed by nitrogen (N_2_) adsorption measurements (Figure 1d), thereby providing more adsorption sites for small metabolites.

Moreover, there is a slight difference in composition between the APF-sphere and APF-bowl. Within 180 min of adding acetone, dissolution of polymer and repolymerization of oligomer continue to occur, which can be explained by the dynamic change of carbon content ratio on the branched-chain (c-^13^C) and benzene ring (r-^13^C) measured by the ^13^C nuclear magnetic resonance (^13^C NMR, Supporting Figure 3a). Notably, the APF-bowl obtained at 180 min have a more significant portion of c-^13^C than APF-sphere (0 min, Supporting Figure 3b). We speculate that the acetone acts as a solvent and participates in the nucleophilic addition and condensation reaction with the hydroxy/amino group of APF.^24^ The above process could also account for the higher content of N and O on the APF-bowl surface than that of the APF-sphere (Supporting Figure 4).

The Au nanoparticles were then introduced for assembling the APF&Au chips by *in situ* reduction of Au precursor on APF with proper reducibility. The APF-sphere&Au chips displayed a rough surface by SEM and transmission electron microscopy (TEM) (Figure 1e,i) with the Au content characterized by energy-dispersive X-ray spectroscopy (EDS) (Supporting Figure 4a–c). The Au nanoparticles were also decorated on the APF-bowl, yielding a series of APF-bowl&*M*Au chips (*M* = 0.72/0.96/1.20) with surface roughness and nanocavities, revealed by electron microscopy images (Figure 1, f to h, and j to l) and atomic force microscopy (AFM) images (Supporting Figure 5). As the Au precursor supply increased, the relative Au contents showed an increasing trend according to the EDS characterizations (Supporting Figure 4d–h). Thermogravimetric (TG) analysis was further carried out to determine the Au content in APF-bowl&*M*Au (Supporting Figure 6), which showed high consistency with only a slight difference in the theoretical value. The uniform distribution of elements in APF-bowl&Au is exhibited in Figure 1m. The crystalline structure of Au nanoparticles in APF-bowl&Au is illustrated by the selected area electron diffraction (SAED) pattern (Figure 1n), high-resolution TEM (HRTEM, Figure 1o), and X-ray diffraction (XRD) pattern (Supporting Figure 7). The stable crystalline structure of the APF-bowl&Au can avoid introducing the background noise under laser irradiation and is desirable for application as an LDI MS matrix. Notably, the valence states of Au in APF-bowl&Au are the mixed states of Au^+^ and Au^0^, characterized by X-ray photoelectron spectroscopy (XPS), while APF-sphere&Au contain an extra small amount of Au^3+^, correlating with the incomplete self-reduction process of the intrinsic APF-sphere (Figure 1p and Supporting Figure 8). The metallic Au in APF-bowl&Au is expected to have better light-trapping ability. As the surface roughness can be regulated to satisfy diverse biomedical applications (Supporting Table 1), the optimized Au loading content with desirable surface nanocavities would strengthen the APF&Au chips regarding the charge transfer as LDI MS matrix for metabolite detection.

Notably, the APF-bowl&Au displays distinct features compared to previously reported noble metal nanostructures in material design and potential in LDI MS detection. From the point of view of material design, the features rely on the following aspects: (1) different from most isotropic structures of noble metal decorated nanostructures (*e.g.*, sphere^9,35^), it is an anisotropic open bowl-structured submicroreactor that we developed, which provides specific micro/nanostructures with enhanced mass diffusion and enrichment of biomarkers for LDI MS application; (2) considering the synthetic method, we prepared APF bowls using a facile strategy of solvent-assisted repolymerization by taking advantage of the difference of polymerization degree of APF, which is more cost-effective than hard-template methods or lithography;^36−38^ (3) for the loading of Au nanoparticles, the Au nanoparticles can be *in situ* reduced in the APF-bowl because of its abundant reducing groups, free of the reductants required in other studies.^39,40^

For LDI MS application, the APF-bowl&Au submicroreactor exhibits great potential in untargeted metabolic detection as compared with most anisotropic morphologies that have been applied for targeted molecule detection (*e.g.*, endogenous metabolites and drug metabolites) (Supporting Table 2). This is attributed to the following aspects: (1) it is simple to adjust the submicroreactor structure (bowl/sphere) and composition (Au loading contents), which is of significance to explore the LDI process and further exploit the MS detection performance; (2) the open bowl-shaped submicroreactor displays an enhanced light-trapping property and large specific surface area, which would be beneficial for harvesting the laser energy and exposing Au nanoparticles to small molecule metabolites (ultraviolet–visible (UV–vis) absorption spectra, Supporting Figure 9);^27^ (3) Au nanoparticles present high production yields of hot charge carriers for expediting the charge transfer on the analyte–matrix interface toward metabolite detection, the tailored loading content of which can modulate the surface chemistry for selective metabolite enrichment.^39,41^

### Exploration of APF&Au Chips for LDI MS Detection

**Figure 2:**
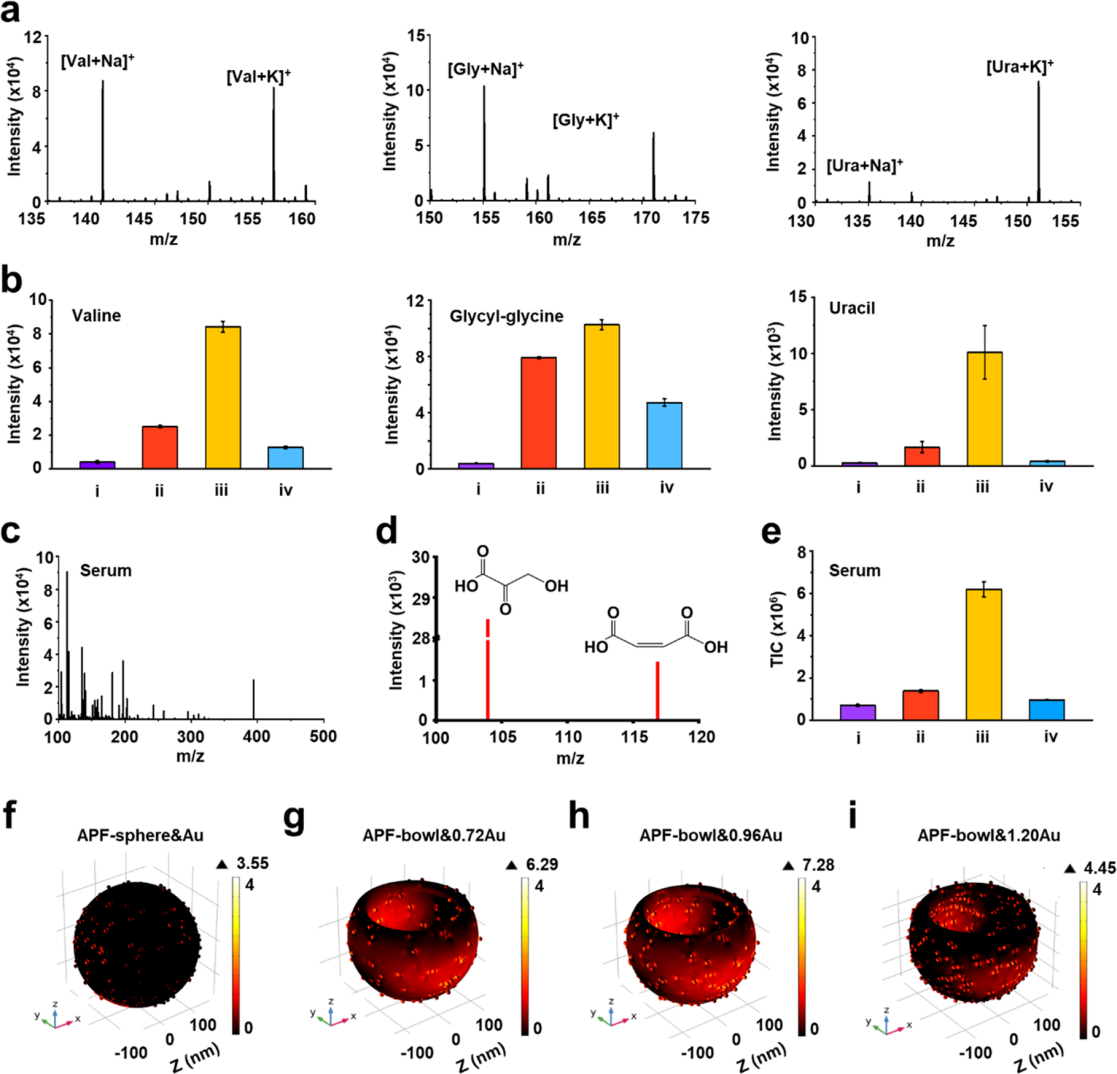
Exploration of APF&Au chips for LDI MS detection. (a) Typical mass spectra using APF-bowl&0.96Au for detecting standard small metabolites of valine (Val), glycyl-glycine (Gly), and uracil (Ura). The [M + Na]^+^ and [M + K]^+^ are separately labeled. (b) Mean signal intensities based on three independent experiments for [M + Na]^+^ of standard small metabolites by using (i) APF-sphere&Au, (ii)APF-bowl&0.72Au, (iii) APF-bowl&0.96Au, and (iv) APF-bowl&1.20Au. (c) The representative mass spectrum from a serum sample using APF-bowl&0.96Au (*m*/*z* of 100–500). (d) Two small metabolites of hydroxybutyric acid (*m*/*z* of 103.95) and maleic acid (*m*/*z* of 116.84) detected in the representative mass spectrum, reported as the biomarkers for ovarian cancer. (e) The averaged TIC for a serum sample based on three independent experiments, by using the matrix of (i) APF-sphere&Au, (ii)APF-bowl&0.72Au, (iii) APF-bowl&0.96Au, and (iv) APF-bowl&1.20Au. The plots of EM field amplitudes for (f) APF-sphere&Au, (g) APF-bowl&0.72Au, (h) APF-bowl&0.96Au, and (i) APF-bowl&1.20Au. The EM field is shown as log(|*E*|^2^/|*E*_0_|^2^), where *E* and *E*_0_ refer to the enhanced field and incident laser, respectively. The results of panels f–i were obtained by the simulation of finite element method with laser wavelength of 355 nm injected along the *z*-axis and laser beam polarized along the *x*-axis.

The APF&Au chips with the adjustable structure of bowl/sphere and tunable Au loading content were applied as matrices for LDI MS detection, including small metabolites and actual serum sample (Figure 2). We used the APF&Au chips for LDI MS detection of typical small metabolites (Figure 2a), yielding the sodium-/potassium-adducted metabolic signals ([M + Na]^+^ and [M + K]^+^) for valine (Val), glycyl-glycine (Gly), and uracil (Ura). Notably, the APF-bowl&0.96Au afforded the highest intensities compared with optimized APF-sphere&Au (APF-sphere&0.96Au, Supporting Figure 10) and APF-bowl loaded with other Au loading content (APF-bowl&0.72Au and APF-bowl&1.20Au) (Figure 2b and Supporting Figure 11). Similar results were also obtained in other representative small metabolites (such as glucose, decanoic acid, and leucine; Supporting Table 3).

Moreover, the series of APF&Au chips were exerted to detect a complex serum sample, and the detection performance could be evaluated by the total ion count (TIC, as the intensity summation of the mass spectrum). The typical mass spectrum of serum, containing ∼120 000 data points (*m*/*z* range of 100–1000), was recorded by APF-bowl&0.96Au assisted LDI MS using only 1 μL of raw serum within 1 min (Figure 2c), affording the TIC of 6.2 × 10^6^ according to three independent measurements. Two ovarian cancer biomarkers of hydroxybutyric acid^42,43^ and maleic acid^44,45^ were detected in the representative mass spectrum, revealing the potential of small metabolites in decoding disease (Figure 2d). In contrast, suboptimal TICs of serum mass spectra were afforded by APF-sphere&Au (TIC of 7.0 × 10^5^), APF-bowl&0.72Au (TIC of 1.4 × 10^6^), and APF-bowl&1.20Au (TIC of 9.6 × 10^5^, *p* < 0.05; Figure 2e and Supporting Table 4). Therefore, the superiority of APF-bowl&0.96Au was demonstrated in both LDI MS detection of small metabolite and serum.

To further exemplify the intrinsic feature of APF-bowl&0.96Au chip as LDI MS matrix, we investigated the localized electromagnetic (EM) field distribution using the finite element method. The incident laser wavelength for simulation was set at 355 nm, in keeping with the laser used for LDI MS, and the size and morphology of the submicroreactor chips refer to the electron microscopy characterizations. The Au nanoparticle of all submicroreactor chips was set as 10 nm to ensure more accurate comparison, the uniform random distribution of which was consistent with the practical synthesis reaction system (Supporting Figure 12). For the structure, the bowl-shaped submicroreactor chips (APF-bowl&Au) owned the higher relative enhancement of 4.45–7.28 in the EM field, higher than that reported for the sphere structure (APF-sphere&Au of 3.55, Figure 2f). The EM field enhancement of the matrix structure correlates with its capability to trap light (*e.g.*, scattering and reflection) for affecting the LDI efficiency.^24,25^ Compared with the spherical architecture, the bowl architecture affords a higher scattering/reflection effect in its concave domain for enhancing EM field and facilitating the LDI process when used as the matrix.^27^ Coupled with the higher specific surface area (Figure 1d), the APF-bowl&Au exhibited the metabolite signals with higher intensities over APF-sphere&Au (Figure 2a–e). Notably, while the bowl-shaped matrix has been explored in surface catalysis, it is rarely employed in metabolic detection. We undertook a preliminarily study to determine the better performance of APF-bowl&Au over APF-sphere&Au according to the LDI MS detection and EM field simulation.

In parallel, we examined the localized EM field with adjustable Au loading content. The APF-bowl&0.96Au displayed the highest relative enhancement of 7.28 (Figure 2h), compared with APF-bowl&0.72Au (relative enhancement of 6.29, Figure 2g) and APF-bowl&1.20Au (relative enhancement of 4.45, Figure 2i). The matrix composition is also crucial to LDI efficiency by altering the localized EM field.^46^ Compared to pure APF-bowl, the introduction of Au nanoparticles on the APF-bowl expands the light adsorption range because of the intrinsic plasmonic effect of Au nanoparticles as a characteristic plasmonic adsorption peak of Au nanoparticles appears at about 600 nm in UV–vis absorption spectra (Supporting Figure 9). Moreover, for the hybrid matrix containing noble metal elements, the loading content can manipulate its distribution and physicochemical properties; the tailored loading content of noble metal elements can adjust the surface roughness and hot carrier yield to generate an enhanced localized EM field (*i.e.*, plasmonic hot spots).^47^ When comparing the different Au loadings, the APF-bowl&0.96Au exhibited superior capability in small metabolite detection (Figure 2a–e) because of its desirable distribution of nanoscaled cavities within Au nanoparticles for higher yield of hot carriers, in line with the EM field simulation results (Figure 2g–i). In addition, we demonstrated the optimized Au loading content of APF-bowl&0.96Au contributes to the size-exclusive effect for selective trapping of small metabolites and excluding the macromolecules (*e.g.*, proteins), displaying the preferable protein tolerance in small metabolite detection (Supporting Figures 13 and 14). Taken together, we demonstrated the specific bowl structure and optimized Au loading content both contribute to APF-bowl&0.96Au as the primary candidate for the construction of the high-performance submicroreactor toward biomedical applications, such as monitoring of cancer treatment.

### Machine Learning of SMFs for Chemotherapy Monitoring

**Figure 3:**
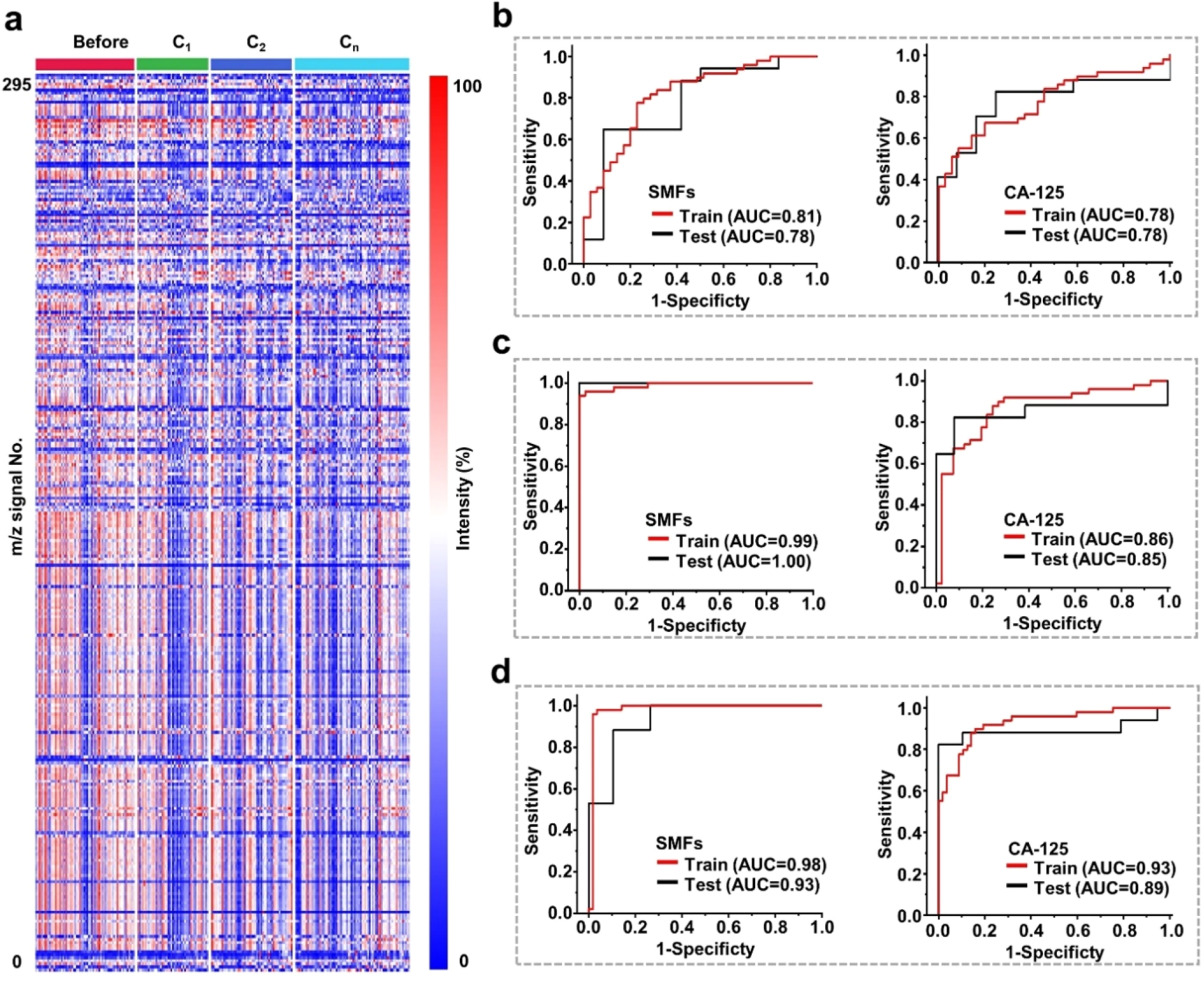
Machine learning of SMFs for chemotherapy monitoring. (a) The blueprint of 243 SMFs, which were extracted from 66 samples before chemotherapy (marked as before), 47 samples after cycle 1 of chemotherapy (marked as C_1_), 54 samples after cycle 2 of chemotherapy (marked as C_2_), and 76 samples after cycle *n* (*n* ≥ 5) of chemotherapy (marked as C_*n*_). Each SMF consisted of 295 *m*/*z* signals by preprocessing the original mass spectra. (b) Differentiation of ovarian patients before chemotherapy and patients in C_1_ group using SMFs (left) and CA-125 (right), respectively. (c) Differentiation of ovarian patients before chemotherapy and patients in C_2_ group using SMFs (left) and CA-125 (right), respectively. (d) Differentiation of ovarian patients before chemotherapy and patients in C_*n*_ group using SMFs (left) and CA-125 (right), respectively. The training and test set ROC curves are presented in red and black with corresponding AUC values labeled.

We extracted the SMFs of ovarian cancer patients before and after different chemotherapy cycles for analysis using the established submicroreactor of APF-bowl&0.96Au chip (Figure 3). We enrolled 243 serum samples of ovarian cancer patients, including 66 samples before chemotherapy, 47 samples after cycle 1 of chemotherapy (marked as C_1_), 54 samples after cycle 2 of chemotherapy (marked as C_2_), and 76 samples after cycle *n* of chemotherapy (*n* ≥ 5, marked as C_*n*_). Each cycle represented a standard chemotherapy treatment of 21 days. All the ovarian cancer patients in this study were diagnosed by pathological examination and received the combined chemotherapy of paclitaxel and carboplatin (Supporting Table 5). According to the Helsinki Declaration, this study was approved by the institutional ethics committees of the Renji Hospital and School of Biomedical Engineering, Shanghai Jiao Tong University (Ethic number 2018-114), and all individuals provided written informed consent to participate in the study and approved the use of their biological samples for analysis.

We recorded the metabolic *m*/*z* signals of ovarian cancer patients before and after different cycles of chemotherapy in the low mass range (*m*/*z* of 100–1000) by using only 1 μL of serum per sample. The original mass spectra of each serum sample (containing ∼120 000 data points) could be obtained in 1 min without tedious pretreatment (Supporting Figure 15) because of the synergetic design of APF-bowl&0.96Au chips for small metabolite enrichment under the interference of salts/macromolecules. On the basis of the desirable submicroreactor of the APF-bowl&0.96Au chip, we extracted SMFs from the above 243 ovarian cancer patients by preprocessing the original mass spectra, yielding 295 *m*/*z* signals for each SMF. Specifically, the 243 SMFs with 295 *m*/*z* signals were further organized as the blueprint in Figure 3a, serving as the database for building a chemotherapy monitoring model.

The power analysis^48,49^ was also conducted to evaluate the sample size of this study (Supporting Figure 16), which was larger than the required minimum number (16 samples per group) for building a machine learning model. The required small sample size in the study is attributed to both the significant metabolic alternations raised by chemotherapy and the superiority of the detection platform. Specifically, the chemotherapy process can significantly change the pathological and physiological conditions of the human body, particularly for small metabolites in the downstream pathways. The collaborative design of the APF-bowl&0.96Au submicroreactor was also critical to extracting the SMFs efficiently, enabling the revelation of metabolic differences during chemotherapy. In addition, the sample size of this study (243 samples) was comparable with that of previous literature (∼30–200 samples).^9,50,51^

We built the chemotherapy monitoring model of ovarian cancer by machine learning of the SMF database and evaluated the model performance by comparing it with a clinical biomarker of CA-125.^10,11^ The monitoring model was built according to the different cycles of chemotherapy. For C_1_, the SMFs achieved the AUC of 0.81 with sensitivity/specificity of 0.78/0.77 for differentiating the patients before chemotherapy, comparable with that of CA-125 (AUC of 0.78 with sensitivity/specificity of 0.65/0.80; Figure 3b and Supporting Table 6). As chemotherapy progressed to cycle 2 and cycle *n* (*n* ≥ 5), the SMFs afforded the enhanced AUC of 0.99 (sensitivity/specificity of 0.96/0.96; Figure 3c and Supporting Table 7) and 0.98 (sensitivity/specificity of 0.98/0.96; Figure 3d and Supporting Table 8), respectively. In contrast, the clinical biomarker of CA-125 exhibited the suboptimal AUC as 0.86 (sensitivity/specificity of 0.86/0.76, Figure 3c) and 0.93 (sensitivity/specificity of 0.86/0.86, Figure 3d). Moreover, we reached similar results in the independent test set, further validating the superior performance of SMFs generated by LDI MS over CA-125, particularly for C_2_ and C_*n*_.

In detail, we examined four primary machine learning methods for reaching satisfactory results, including elastic net (EN), least absolute shrinkage and selection operator (LASSO), partial least-squares (PLS) regression, and decision tree (Supporting Figures 17–19 and Supporting Tables 6–8). The study design of training and independent test sets can avoid overfitting performance, thus obtaining a robust chemotherapy monitoring model. Considering the rational design of the model building, we could conclude that SMFs outperformed the current clinical biomarker of CA-125 in chemotherapy monitoring according to diagnostic performance in both training and test sets.

Toward the clinical application on a large scale, the submicroreactor for SMFs also needs to address sensitivity, throughput, and analytical speed requirements. Accordingly, the APF-bowl&0.96Au assisted LDI MS provides the following advantages: (1) high sensitivity at femtomolar level due to the advanced submicroreactor of APF-bowl&0.96Au in metabolic signal detection, superior to the widely used NMR with sensitivity at the micromolar level;^13,14^ (2) high throughput, as the original mass spectrum recorded by APF-bowl&0.96Au contains ∼120 000 data points and 295 *m*/*z* signals after processing (Figure 3a); and (3) fast analytical speed of <1 min per sample for mass spectrum acquisition due to the direct serum analysis in a label-free manner with no tedious pretreatment (*e.g.*, derivatization).^52^ Hence, the APF-bowl&0.96Au assisted LDI MS can achieve efficient SMF extraction, serving as an ideal submicroreactor for chemotherapy monitoring in the clinical scenario.

Clinical chemotherapy monitoring is guided by the response evaluation criteria in solid tumors version 1.1 (RECIST v1.1), which is assisted with CT and magnetic resonance imaging (MRI) imaging tools for characterizing the tumor lesions within the treatment period.^5^ However, RECIST v1.1 cannot satisfy the clinical needs in chemotherapy monitoring because of the heterogeneity of tumor growth, delayed feedback, and suboptimal resolution of imaging tools.^6^ In addition, no single tumor biomarker could be applied for chemotherapy evaluation because of the limited monitoring performance. Although the CA-125 was reported as the diagnostic and prognostic biomarker of ovarian cancer, its serum level showed restriction in the clinical use of chemotherapy monitoring, particularly for ovarian cancer patients with low serum levels of CA-125 before chemotherapy. As illustrated by our results, the SMFs outperformed CA-125 in chemotherapy monitoring through the accurate profiling of human physiological states with different cycles of chemotherapy (Figure 3b–d). Moreover, compared to imaging tools, the established submicroreactor also affords in-time feedback and high resolution (within ±10 mDa), thus demonstrating its potential as a next-generation tool for chemotherapy monitoring.

### Potential Biomarkers for Chemotherapy Monitoring

**Figure 4:**
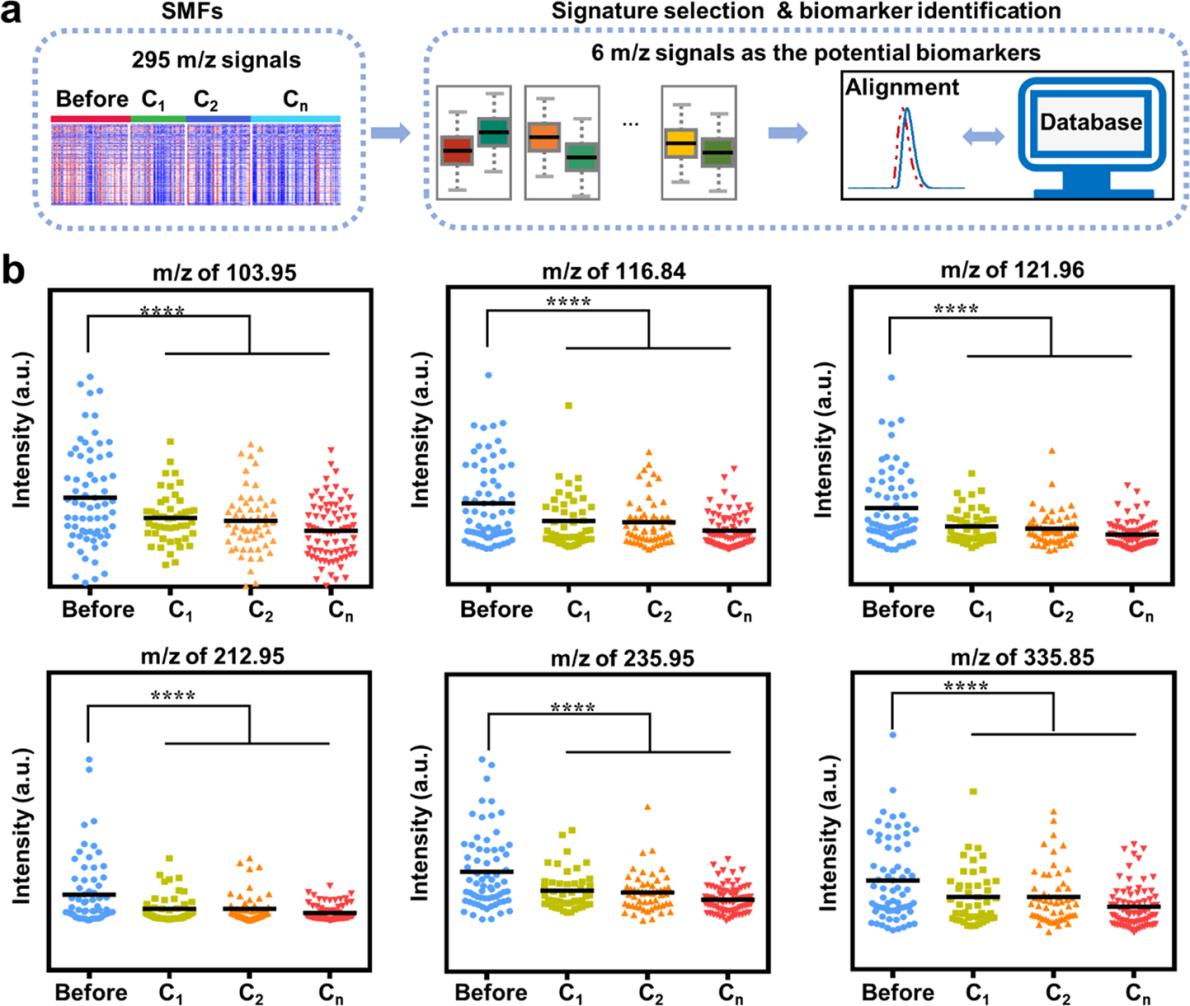
Potential biomarkers for chemotherapy monitoring. (a) The workflow for screening out the potential biomarkers from SMFs of 295 *m*/*z* signals. For signature selection, six *m*/*z* signals were screened out with significant differences before and after chemotherapy of ovarian cancer patients. For biomarker identification, six potential biomarkers were identified by accurate mass measurements and alignment with the metabolite database. (b) Scatter plots of potential biomarkers among ovarian patients before chemotherapy and after chemotherapy of C_1_/C_2_/C_*n*_ (*n* ≥ 5). The significant difference between ovarian patients before and after chemotherapy was measured by the statistical test, and a gradual decrease trend was found as the chemotherapy cycle proceeded. **** indicates *p***<** 0.0001.

We preliminarily identified a metabolic biomarker panel for chemotherapy monitoring, including six metabolites selected from the SMFs of ovarian cancer patients (Figure 4; for more details, see Methods). In addition to SMF extraction from the raw mass spectrum by data processing, two main procedures for screening out potential metabolic biomarkers were signature selection and biomarker identification (Figure 4a). Specifically, for the signal selection, we screened out six *m*/*z* signals with a significant difference (*p* < 0.05) between ovarian cancer patients before chemotherapy and after chemotherapy (Figure 4b). For biomarker identification, six potential biomarkers were obtained by accurate mass measurements and alignment with the human metabolite database (Supporting Table 9, see more details in Methods). As chemotherapy proceeded, these six potential biomarkers showed a gradual decreasing trend (Figure 4b and Supporting Table 10).^53^

The biomarker with related pathway analysis was also conducted using MetaboAnalyst (http://www.metaboanalyst.ca/).^48,54^ For biomarker analysis, we evaluated the fold change of potential biomarkers before and after chemotherapy based on the corresponding signal intensities. Specifically, the most significant fold change of 2.58 and lowest fold change of 1.43 were afforded by N-acetylasparagine and hydroxybutyric acid, respectively (Supporting Table 10). We identified two altered pathways related to the above biomarker panel for pathway analysis: the glyoxylate and dicarboxylate metabolism (pathway impact of 0.22) and glycine, serine, and threonine metabolism (pathway impact of 0.05, Supporting Figure 20). Alternation of these two pathways resulted from the downregulation of hydroxybutyric acid after chemotherapy. The above pathway analysis based on the constructed biomarker panel would provide more insights for unveiling chemotherapy monitoring.

The metabolic biomarker serves as the indicator of biological conditions and is critical to the illustration of chemotherapy mechanism.^55,56^ Specifically, four metabolic biomarkers identified in this study showed high consistency with previous literature: For hydroxybutyric acid, it has been reported as the diagnostic and prognostic biomarkers of ovarian high-grade serous carcinomas, the serum level of which may indicate cancer cell migration and invasion and contribute to chemotherapy monitoring.^42,43^ Maleic acid can affect chemotherapy efficiency by altering the tricarboxylic acid cycle and the tumor cell energy metabolism.^44,45^ Notably, cysteine released by fibroblasts leads to platinum-based chemotherapy resistance, the decreased level of which can predict an efficient chemotherapy.^57,58^ For N-acetylasparagine, its decreased level correlates with the depletion of aspartate toward inhibition of tumor growth.^59^ 3-Hydroxy-2-methylpyridine-4,5-dicarboxylate and dihydroneopterin phosphate are two metabolic biomarkers first discovered in this study, presumably affecting the chemotherapy *via* vitamin B6 metabolism and biosynthesis of folate.^60,61^ Regarding the crucial role of metabolites in the pathway analysis, the established biomarker panel can guide targeting and efficient cancer chemotherapy.

## Conclusion

In conclusion, we fabricated the submicroreactor of APF-bowl&Au chips for recording the SMFs by LDI MS and achieved rapid and precise chemotherapy monitoring with the assistance of machine learning methods. The APF&Au chips with anisotropic bowl structure and preferable Au loading content have been well investigated, affording the enhanced EM field for efficient LDI MS detection and SMFs extraction. Machine learning of the SMFs achieved the chemotherapy monitoring of ovarian cancer with an AUC of 0.81–0.98 and yielded a metabolic biomarker panel, promoting the revelation of the chemotherapy mechanism. Our advanced detection platform based on the bowl-structured submicroreactor chips would efficiently monitor cancer chemotherapy and is very promising to be a universal tool for clinical application on a large scale.

The limitations and future research lines of this study need to be stated as follows: (1) our detection platform relies on the MS system for recording the SMFs, which may restrict its potential use from POCT; (2) the investigation of anisotropic structures beyond bowl-shaped matrix would contribute to an elevated LDI efficiency and improved metabolic analysis; (3) a comprehensive evaluation of chemotherapy efficiency would be achieved by involving more individuals with follow-up visit outcomes; (3) a well-designed case-cohort with cancer patients besides ovarian cancer could further increase the performance and extend the application scenarios of our platform.

## Methods

### Chemicals and Reagents

Formaldehyde solution (CH_2_O, 36.0%) was obtained from Aladdin Reagent (Shanghai, China). 3-Aminophenol, trifluoroacetic acid (TFA, 99%), valine (98%), glycyl-glycine (99%), uracil (99%), glucose (99.5%), decanoic acid (98%), leucine (98%), methionine (98%), and bovine serum albumin (BSA) were obtained from Sigma-Aldrich (St. Louis, MO, United States). The acetone (99.5%), ammonia aqueous solution (NH_3_·H_2_O, 28%), and chloroauric acid tetrahydrate (HauCl_4_·4H_2_O, 47.8%) were purchased from Sinopharm Chemical Reagent Co., Ltd. (Beijing, China). Deionized water (18.2 MΩ cm, Milli-Q, Millipore, GmbH) was utilized for preparing the aqueous solution in this study.

### Preparation of APF-sphere and APF-bowl

The APF-sphere was fabricated by polymerization of the formaldehyde and 3-aminophenol. Typically, 3-aminophenol (0.1 g) was dissolved in 30 mL of deionized water, followed by the addition of formaldehyde solution (0.1 mL) and aqueous ammonia solution (0.1 mL). The mixture was stirred at 30 °C for 30 min, yielding APF-sphere after washing with deionized water 5 times. The APF-bowl was synthesized by dissolving the APF-sphere with acetone solution. In detail, the acetone solution (40 mL) was directly added into the APF-sphere suspension that was already polymerizing for 30 min, and the mixture was then stirred at 30 °C for 3 h. The APF-bowl could be obtained by washing the mixture with deionized water and drying it at 100 °C for 6 h.

### Preparation of APF&Au Chips

The APF-sphere (10 mg) was dissolved in 10 mL of deionized water, followed by the addition of 1.5/2.0/2.5 mL of HauCl_4_ aqueous solution (1%). The mixture was stirred at 70 °C for 10 min and washed with deionized water 5 times to obtain APF-sphere&*M*Au. The HAuCl_4_ aqueous solution (1%) supply of 2.0 mL resulted in the optimized Au loading content of the APF-sphere&Au. Similar procedures were conducted for getting the chips of APF-bowl&*M*Au. The formula for calculating *M* is as follows:
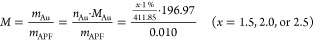
1*M* of 0.72/0.96/1.20 corresponds to 1% HauCl_4_ aqueous solution usage of 1.5/2.0/2.5 mL, respectively.

### Characterization Methods

SEM images and EDS were obtained by a Hitachi S-4800 instrument (Hitachi, Japan). TEM images, HR TEM, and SAED were recorded by the JEOL JEM-2100F instrument. In addition to the EDS that was used to monitor the changing trend of Au content (APF-bowl&0.72Au, APF-bowl&0.96Au, and APF-bowl&1.20Au), TG analysis was further conducted to match calculated theoretical content using a PerkinElmer TGA4000 instrument under air flow. The ultraviolet–visible (UV–vis) absorption spectra were recorded by a UV2700 instrument (Shimadzu, Japan). The elemental mapping characterizations were performed by a Talos F200X G2 apparatus. For further illustrating the size-exclusive effect, the APF-bowl&0.96Au submicroreactor was physically mixed with methionine (10 mg/mL) with a volume ratio of 1/1 and incubated for 30 min. The residues after centrifugation were redispersed in deionized water for analysis. The XRD pattern was recorded by Rigaku D/Max 2500/PC. The XPS was obtained from a KRATOS Axis Ultra^DLD^ apparatus by using Al ka (hl 1/4 1486.6 eV) as the excitation light source. The specific surface area and N_2_ adsorption–desorption isotherms were characterized by an ASAP 2460 Micropore Physisorption Analyzer. Solid-state ^13^C NMR spectra were measured on a Bruker Avance III 600 MHz spectrometer with a 3.2 mm DVT MAS probe and a spinning rate of 20 kHz. The AFM was conducted on the Asylum MFP-3D by dropping the aqueous solution of APF-bowl&*M*Au on the silica wafer.

### Serum Sample Characteristics

A total of 243 serum samples from ovarian cancer patients were collected from Renji Hospital, School of Medicine, Shanghai Jiao Tong University. All the patients were diagnosed according to pathological examination and received the combined chemotherapy of paclitaxel and carboplatin. The disease stage of each ovarian cancer patient was obtained according to the standards of International Federation of Gynecology and Obstetrics (FIGO) 2018 for ovarian cancer. There were 66 serum samples collected before chemotherapy, 47 serum samples collected after cycle 1 of chemotherapy (C_1_), 54 serum samples collected after cycle 2 of chemotherapy (C_2_), and 76 serum samples collected after cycle *n* (*n* ≥ 5) of chemotherapy (C_*n*_). Each cycle represented the chemotherapy treatment of 21 days, according to clinical standards for cancer. The serum samples were all harvested according to the established standards^62^ and stored at −80 °C in the refrigerator before use. This study was approved by the institutional ethics committees of the Renji Hospital and School of Biomedical Engineering, Shanghai Jiao Tong University (Ethic number of 2018-114). According to the Helsinki Declaration, all individuals provided written informed consents to participate in the study and approved the use of their biological samples for analysis.

### LDI MS Detection

In this study, the LDI MS analysis was conducted on the positive ion mode of MALDI-TOF/TOF mass spectrometry (Bruker) equipped with both a Nd:YAG laser (2 kHz, 355 nm) and smart beam system. The parameter settings included a repetition rate of 1 kHz, acceleration of 20 kV, a delay time of 150 ns, and laser shots of 2000 per analysis. Notably, the accurate mass calibration within ±10 mDa was achieved by using standard small molecules. Typically, 1 μL of analyte solution was mixed with 1 μL of matrix suspension for LDI MS detection. For the analyte solution, the standard small metabolites (including valine, glucose, decanoic acid, glycyl-glycine, uracil, and leucine) were dissolved in deionized water with the concentration of 1 ng/nL; the serum samples were diluted with deionized water by 10 fold. For the protein tolerance, the BSA (5 mg/mL) was separately mixed with methionine (1 ng/nL) and the mixture of typical small metabolites (including valine, glycyl-glycine, and uracil, each at the concentration of 1 ng/nL). For matrix suspension, the particles of APF-sphere&Au and APF-bowl&Au were dispersed as the aqueous solution of 1 ng/nL. In addition, the blank control, APF-sphere, and APF-bowl were prepared with the same protocol as APF&Au chips for LDI MS.

### Simulation by Finite Element Method

We compared the optical responses of APF&Au chips in terms of core structure (APF-sphere and APF-bowl) and Au shell content using the finite element method (FEM). The FEM within the Wave Optical Module of COMSOL Multiphysics can solve the Holmholtz equation about the time-harmonic electric field (*E*):
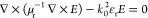
2where *k*_0_ is the wave vector; *ε*_r_ = (*n* + i*k*)^2^ is the relative permittivity; *μ*_r_ = 1 is the relative permeability; and *n* and *k* are the real and imaginary parts of the complex refractive index, respectively.^63^ The refractive index of Au is modeled according to the linear interpolation of experimental data from Johnson and Christy. The refractive index of the APF was set as 1.555, and the refractive index of the surrounding air could be set as 1. The size and morphology of nanoparticles were characterized by electron microscopy characterizations. The Au nanoparticle sizes of APF-sphere&Au and APF-bowl&*M*Au (M = 0.72/0.96/1.20) were set as 10 nm, ensuring the validity of simulation results for comparison. The space of neighboring Au nanoparticles was determined by their distribution on the APF-bowl/APF-sphere. The uniform random distribution was adopted for Au nanoparticles in APF-bowl/APF-sphere, consistent with the practical liquid reaction system. The space between neighboring Au nanoparticles is around 25.8 nm (observed from the APF-bowl&0.72Au when the Au nanoparticles were of uniform distribution). A plane incident light wave at 355 nm was set along the negative *z*-axis and polarized along the *x*-axis with an amplitude of 1 V/m, according to the laser wavelength equipped in LDI MS. The calculated region was surrounded by a perfectly matched layer (PML) with a spherical shape. The relationship between electric field intensity (*I*) and electric field (*E*) is
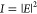
3

### Machine Learning-Assisted Chemotherapy Monitoring

Four machine learning algorithms were applied to the previously extracted SMFs, including three for linear modeling and one for nonlinear modeling. For linear modeling, elastic net,^64^ the least absolute shrinkage and selection operator (LASSO),^65^ and partial least-squares (PLS) regression^66^ were included. From the aspect of sparsity analysis, elastic net was regularized from logistic regression with *l*_1_-norm and the squared *l*_2_-norm:
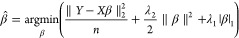
4and LASSO with *l*_1_-norm was obtained by the following formula:
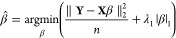
5where λ_1_ ≥ 0 and λ_2_ ≥ 0 control L1 and L2 regularization, *n* is the sample number, **X** is the matrix for SMFs, and **Y** is the vector for clinical outcomes of interest (*e.g.*, “0” for patients before chemotherapy and “1” for patients in cycle 1). In the aspect of multicollinearity analysis, PLS regression with latent variables projection was included. The PLS formula that we used is as follows:
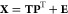
6
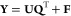
7where **P** and **Q** are the loading matrix, **T** and **U** are the projections of **X** and **Y**, and **E** and **F** are the residual matrices, respectively. The decomposition of **X** and **Y** are trained to maximize the covariance between **T** and **U**. For nonlinear modeling, decision tree^67^ with cascaded if–then–else rules was compared, and we solved the overfitting problem by optimizing the minimum number of samples required to be at a leaf node. The best minimum number was tuned from 1 to 50 with a step of 1. All classification algorithms were trained by a 5-fold cross-validation strategy and ran under the same environment (python 3.7.4 and scikit-learn 0.23.2). The PLS, LASSO, and EN were for differentiating the before the chemotherapy from the C_1_ group, C_2_ group, and C_*n*_ group, respectively, considering the diagnostic performance in both training and test sets. In addition, as a sufficient sample number is critical to building a robust machine learning model, the power analysis can be conducted *via* Metaboanalyst^48^ to evaluate the sample size. The threshold of predicted power can be set as 0.8 according to previous literature reports.^55^

### Potential Biomarker Identification

The potential biomarker was identified according to the established procedures. First, the SMFs of 295 *m*/*z* signals were obtained by preprocessing of the original MS spectra with ∼12 000 data points. Then, the specific *m*/*z* signals, which showed significant differences between patients before and after chemotherapy, were screened out for further analysis. Importantly, the potential biomarkers corresponding to the specific *m*/*z* signals could be identified by accurate mass measurements and alignment with the human metabolite database (https://hmdb.ca/). The six metabolic biomarkers are hydroxybutyric acid, maleic acid, d-cysteine, N-acetylasparagine, 3-hydroxy-2-methylpyridine-4,5-dicarboxylate, and dihydroneopterin phosphate.

### Statistical Analysis

One-way ANOVA analysis^68^ was conducted for comparing the detection performance of APF&Au chips in small-molecule detection and total ion count (TIC) in a representative serum sample. The TIC can be calculated as the summation of intensities in the mass spectrum. Specifically, the APF&Au chips, including APF-sphere&Au, APF-bowl&0.72Au, APF-bowl&0.96Au, and APF-bowl&1.20Au, were applied for small-molecule detection with three independent experiments conducted. The *t* test was used for screening out the potential biomarkers between ovarian cancer patients before chemotherapy and after chemotherapy, involving five independent MS spectra for each serum sample.

## Supporting Information Available

Elemental mapping characterization of APF-sphere (Supporting Figure 1); characterization of APF-bowl obtained by solvent-assisted repolymerization process (Supporting Figure 2); characterization of ^13^C NMR (Supporting Figure 3); EDS characterization of sphere-shaped and bowl-shaped chips (Supporting Figure 4); characterization of AFM (Supporting Figure 5); TG characterization (Supporting Figure 6); XRD pattern of APF-bowl&0.96Au (Supporting Figure 7); XPS characterization (Supporting Figure 8); UV–vis absorbance spectra (Supporting Figure 9); bar graph of LDI MS detection (Supporting Figures 10 and 11); FDTD of APF-bowl&0.96Au (Supporting Figure 12); protein tolerance of APF-bowl&0.96Au in LDI MS detection (Supporting Figure 13); elemental mapping characterizations (Supporting Figure 14); typical MS spectra of ovarian cancer patients before and after chemotherapy (Supporting Figure 15); power analysis (Supporting Figure 16); machine learning of serum metabolic fingerprints (SMFs) for differentiating the ovarian cancer patients before chemotherapy with patients in C_1_ group, C_2_ group, and C_*n*_ group (Supporting Figures 17–19); pathway analysis (Supporting Figure 20); diverse biomedical applications by regulating surface roughness (Supporting Table 1); comparison between the present APF-bowl&Au with previously reported anisotropic morphologies as LDI MS matrix (Supporting Table 2); intensity of typical small-molecule metabolites by LDI MS detection (Supporting Table 3); TIC of mass spectra from a representative serum sample of an ovarian cancer patient (Supporting Table 4); demographic information and clinical features of ovarian cancer patients (Supporting Table 5); diagnostic parameters by differentiating the patients before chemotherapy with chemotherapy cycle 1 (C_1_), C_2_ group, and C_*n*_ group (Supporting Tables 6–8); potential biomarkers selected from SMFs (Supporting Table 9); statistics of potential biomarkers after variable cycle of chemotherapy (Supporting Table 10) (PDF)

## References

[ref1] Miller K. D.; Nogueira L.; Mariotto A. B.; Rowland J. H.; Yabroff K. R.; Alfano C. M.; Jemal A.; Kramer J. L.; Siegel R. L. Cancer treatment and survivorship statistics, 2019. CA-Cancer J. Clin. 2019, 69, 363–385. 10.3322/caac.21565. 31184787

[ref2] Pfisterer J.; Shannon C. M.; Baumann K.; Rau J.; Harter P.; Joly F.; Sehouli J.; Canzler U.; Schmalfeldt B.; Dean A. P.; Hein A.; Zeimet A. G.; Hanker L. C.; Petit T.; Marme F.; El-Balat A.; Glasspool R.; de Gregorio N.; Mahner S.; Meniawy T. M.; Park-Simon T. W.; Mouret-Reynier M. A.; Costan C.; Meier W.; Reinthaller A.; Goh J. C.; L’Haridon T.; Hay S. B.; Kommoss S.; du Bois A.; Kurtz J. E.; Investig A.-O. E.-o.; et al. Bevacizumab and platinum-based combinations for recurrent ovarian cancer: a randomised, open-label, phase 3 trial. Lancet Oncol. 2020, 21, 699–709. 10.1016/S1470-2045(20)30142-X. 32305099

[ref3] Slamon D. J.; Neven P.; Chia S.; Fasching P. A.; De Laurentiis M.; Im S. A.; Petrakova K.; Bianchi G. V.; Esteva F. J.; Martin M.; Nusch A.; Sonke G. S.; De la Cruz-Merino L.; Beck J. T.; Pivot X.; Sondhi M.; Wang Y. B.; Chakravartty A.; Rodriguez-Lorenc K.; Taran T.; Jerusalem G. Overall Survival with Ribociclib plus Fulvestrant in Advanced Breast Cancer. N. Engl. J. Med. 2020, 382, 514–524. 10.1056/NEJMoa1911149. 31826360

[ref4] Gadgeel S.; Rodriguez-Abreu D.; Speranza G.; Esteban E.; Felip E.; Domine M.; Hui R. N.; Hochmair M. J.; Clingan P.; Powell S. F.; Cheng S. Y. S.; Bischoff H. G.; Peled N.; Grossi F.; Jennens R. R.; Reck M.; Garon E. B.; Novello S.; Rubio-Viqueira B.; Boyer M.; Kurata T.; Gray J. E.; Yang J.; Bas T.; Pietanza M. C.; Garassino M. C. Updated Analysis From KEYNOTE-189: Pembrolizumab or Placebo Plus Pemetrexed and Platinum for Previously Untreated Metastatic Nonsquamous Non-Small-Cell Lung Cancer. J. Clin. Oncol. 2020, 38, 1505–1517. 10.1200/JCO.19.03136. 32150489

[ref5] Eisenhauer E. A.; Therasse P.; Bogaerts J.; Schwartz L. H.; Sargent D.; Ford R.; Dancey J.; Arbuck S.; Gwyther S.; Mooney M.; Rubinstein L.; Shankar L.; Dodd L.; Kaplan R.; Lacombe D.; Verweij J. New response evaluation criteria in solid tumours: Revised RECIST guideline (version 1.1). Eur. J. Cancer 2009, 45, 228–247. 10.1016/j.ejca.2008.10.026. 19097774

[ref6] Yabuuchi H.; Kawanami S.; Iwama E.; Okamoto I.; Kamitani T.; Sagiyama K.; Yamasaki Y.; Honda H. Prediction of Therapeutic Effect of Chemotherapy for NSCLC Using Dual-Input Perfusion CT Analysis: Comparison among Bevacizumab Treatment, Two-Agent Platinum-based Therapy without Bevacizumab, and Other Non-Bevacizumab Treatment Groups. Radiology 2018, 286, 685–695. 10.1148/radiol.2017162204. 29059037

[ref7] Tan S. K.; Pastori C.; Penas C.; Komotar R. J.; Ivan M. E.; Wahlestedt C.; Ayad N. G. Serum long noncoding RNA HOTAIR as a novel diagnostic and prognostic biomarker in glioblastoma multiforme. Mol. Cancer 2018, 17, 74 10.1186/s12943-018-0822-0. 29558959 PMC5861620

[ref8] Mehra N.; Dolling D.; Sumanasuriya S.; Christova R.; Pope L.; Carreira S.; Seed G.; Yuan W.; Goodall J.; Hall E.; Flohr P.; Boysen G.; Bianchini D.; Sartor O.; Eisenberger M. A.; Fizazi K.; Oudard S.; Chadjaa M.; Mace S.; de Bono J. S. Plasma Cell-free DNA Concentration and Outcomes from Taxane Therapy in Metastatic Castration-resistant Prostate Cancer from Two Phase III Trials (FIRSTANA and PROSELICA). Eur. Urol. 2018, 74, 283–291. 10.1016/j.eururo.2018.02.013. 29500065 PMC6090941

[ref9] Cao J.; Shi X. J.; Gurav D. D.; Huang L.; Su H. Y.; Li K. K.; Niu J. Y.; Zhang M. J.; Wang Q.; Jiang M. W.; Qian K. Metabolic Fingerprinting on Synthetic Alloys for Medulloblastoma Diagnosis and Radiotherapy Evaluation. Adv. Mater. 2020, 32, 2000906 10.1002/adma.202000906. 32342553

[ref10] Henderson J. T.; Webber E. M.; Sawaya G. F. Screening for Ovarian Cancer Updated Evidence Report and Systematic Review for the US Preventive Services Task Force. JAMA-J. Am. Med. Assoc. 2018, 319, 595–606. 10.1001/jama.2017.21421. PMC13335868 29450530

[ref11] Lindemann K.; Kristensen G.; Mirza M. R.; Davies L.; Hilpert F.; Romero I.; Ayhan A.; Burges A.; Rubio M. J.; Raspagliesi F.; Huizing M.; Creemers G. J.; Lykka M.; Lee C. K.; Gebski V.; Pujade-Lauraine E. Poor concordance between CA-125 and RECIST at the time of disease progression in patients with platinum-resistant ovarian cancer: analysis of the AURELIA trial. Ann. Oncol. 2016, 27, 1505–1510. 10.1093/annonc/mdw238. 27407100

[ref12] Huang L.; Wan J.; Wei X.; Liu Y.; Huang J.; Sun X.; Zhang R.; Gurav D. D.; Vedarethinam V.; Li Y.; Chen R.; Qian K. Plasmonic silver nanoshells for drug and metabolite detection. Nat. Commun. 2017, 8, 220 10.1038/s41467-017-00220-4. 28790311 PMC5548796

[ref13] Diehl B. Chapter 1 - Principles in NMR Spectroscopy. In NMR Spectroscopy in Pharmaceutical Analysis; Holzgrabe U., Wawer I., Diehl B., Eds.; Elsevier: Amsterdam, 2008; pp 1–41.

[ref14] Zangoli M.; Di Maria F. Synthesis, characterization, and biological applications of semiconducting polythiophene-based nanoparticles. View 2021, 2, 20200086 10.1002/VIW.20200086.

[ref15] Sun S. Y.; Liu W. S.; Yang J.; Wang H.; Qian K. Nanoparticle-Assisted Cation Adduction and Fragmentation of Small Metabolites. Angew. Chem., Int. Ed. 2021, 60, 11310–11317. 10.1002/anie.202100734. 33629432

[ref16] Samarah L. Z.; Vertes A. Mass spectrometry imaging based on laser desorption ionization from inorganic and nanophotonic platforms. View 2020, 1, 20200063 10.1002/VIW.20200063.

[ref17] Tanaka K.; Waki H.; Ido Y.; Akita S.; Yoshida Y.; Yoshida T.; Matsuo T. Protein and polymer analyses up to m/z 100 000 by laser ionization time-of-flight mass spectrometry. Rapid Commun. Mass Spectrom. 1988, 2, 151–153. 10.1002/rcm.1290020802.

[ref18] Karas M.; Hillenkamp F. Laser desorption ionization of proteins with molecular masses exceeding 10,000 Da. Anal. Chem. 1988, 60, 2299–301. 10.1021/ac00171a028. 3239801

[ref19] Endo K.; Ube H.; Shionoya M. Multi-Stimuli-Responsive Interconversion between Bowl- and Capsule-Shaped Self-Assembled Zinc(II) Complexes. J. Am. Chem. Soc. 2020, 142, 407–416. 10.1021/jacs.9b11099. 31804816

[ref20] Jiang S.; Huang K.; Qu J.; Lin J.; Huang P. Cancer nanotheranostics in the second near-infrared window. View 2021, 2, 20200075 10.1002/VIW.20200075.

[ref21] Wang X.; Huang S. C.; Hu S.; Yan S.; Ren B. Fundamental understanding and applications of plasmon-enhanced Raman spectroscopy. Nat. Rev. Phys. 2020, 2, 253–271. 10.1038/s42254-020-0171-y.

[ref22] Su H.; Liu T.; Huang L.; Huang J.; Cao J.; Yang H.; Ye J.; Liu J.; Qian K. Plasmonic Janus hybrids for the detection of small metabolites. J. Mater. Chem. B 2018, 6, 7280–7287. 10.1039/C8TB01587B. 32254639

[ref23] Kim M.-J.; Yun T. G.; Noh J.-Y.; Song Z.; Kim H.-R.; Kang M.-J.; Pyun J.-C. Laser-Induced Surface Reconstruction of Nanoporous Au-Modified TiO2 Nanowires for In Situ Performance Enhancement in Desorption and Ionization Mass Spectrometry. Adv. Funct. Mater. 2021, 31, 2102475 10.1002/adfm.202102475.

[ref24] Yu R. T.; Huang X. D.; Liu Y.; Kong Y. Q.; Gu Z. Y.; Yang Y.; Wang Y.; Ban W. H.; Song H.; Yu C. Z. Shaping Nanoparticles for Interface Catalysis: Concave Hollow Spheres via Deflation-Inflation Asymmetric Growth. Adv. Sci. 2020, 7, 2000393 10.1002/advs.202000393. PMC7341089 32670764

[ref25] Pei F.; An T. H.; Zang J.; Zhao X. J.; Fang X. L.; Zheng M. S.; Dong Q. F.; Zheng N. F. From Hollow Carbon Spheres to N-Doped Hollow Porous Carbon Bowls: Rational Design of Hollow Carbon Host for Li-S Batteries. Adv. Energy Mater. 2016, 6, 1502539 10.1002/aenm.201502539.

[ref26] Chen J.; Bai Y.; Feng J.; Yang F.; Xu P.; Wang Z.; Zhang Q.; Yin Y. Anisotropic Seeded Growth of Ag Nanoplates Confined in Shape-Deformable Spaces. Angew. Chem., Int. Ed. 2021, 60, 4117–4124. 10.1002/anie.202011334. 33037723

[ref27] Li X. X.; Shang Y.; Lin J.; Li A. R.; Wang X. T.; Li B.; Guo L. Temperature-Induced Stacking to Create Cu2O Concave Sphere for Light Trapping Capable of Ultrasensitive Single-Particle Surface-Enhanced Raman Scattering. Adv. Funct. Mater. 2018, 28, 1801868 10.1002/adfm.201801868.

[ref28] Niihori Y.; Wada Y.; Mitsui M. Single Platinum Atom Doping to Silver Clusters Enables Near-Infrared-to-Blue Photon Upconversion. Angew. Chem., Int. Ed. 2021, 60, 2822–2827. 10.1002/anie.202013725. 33295118

[ref29] Nazemi M.; Panikkanvalappil S. R.; Liao C.-K.; Mahmoud M. A.; El-Sayed M. A. Role of Femtosecond Pulsed Laser-Induced Atomic Redistribution in Bimetallic Au-Pd Nanorods on Optoelectronic and Catalytic Properties. ACS Nano 2021, 15, 10241–10252. 10.1021/acsnano.1c02347. 34032116

[ref30] Chen W.; Roelli P.; Hu H.; Verlekar S.; Amirtharaj S. P.; Barreda A. I.; Kippenberg T. J.; Kovylina M.; Verhagen E.; Martínez A.; Galland C. Continuous-wave frequency upconversion with a molecular optomechanical nanocavity. Science 2021, 374, 1264–1267. 10.1126/science.abk3106. 34855500

[ref31] Zhang Q.; Tian Y.; Liang Z.; Wang Z.; Xu S.; Ma Q. DNA-Mediated Au-Au Dimer-Based Surface Plasmon Coupling Electrochemiluminescence Sensor for BRCA1 Gene Detection. Anal. Chem. 2021, 93, 3308–3314. 10.1021/acs.analchem.0c05440. 33533597

[ref32] Shan B.; Li L.; Zhao Y.; Wang H.; Li M. Near-Infrared II Plasmonic Au@Au-Ag Dot-in-Cubic Nanoframes for In Vivo Surface-Enhanced Raman Spectroscopic Detection and Photoacoustic Imaging. Adv. Funct. Mater. 2021, 31, 2103186 10.1002/adfm.202103186.

[ref33] Bin D. S.; Chi Z. X.; Li Y. T.; Zhang K.; Yang X. Z.; Sun Y. G.; Piao J. Y.; Cao A. M.; Wan L. J. Controlling the Compositional Chemistry in Single Nanoparticles for Functional Hollow Carbon Nanospheres. J. Am. Chem. Soc. 2017, 139, 13492–13498. 10.1021/jacs.7b07027. 28858501

[ref34] Zhou S.; Bai Y.; Xu W.; Feng J.; Wang X.; Li Z.; Yin Y. Formation of resorcinol-formaldehyde hollow nanoshells through a dissolution-regrowth process. Nanoscale 2020, 12, 15460–15465. 10.1039/D0NR01143F. 32666993

[ref35] Huang L.; Gurav D. D.; Wu S.; Xu W.; Vedarethinam V.; Yang J.; Su H.; Wan X.; Fang Y.; Shen B.; Price C.-A. H.; Velliou E.; Liu J.; Qian K. A Multifunctional Platinum Nanoreactor for Point-of-Care Metabolic Analysis. Matter 2019, 1, 1669 10.1016/j.matt.2019.08.014.

[ref36] Liang J.; Chen J.; Shen H. Q.; Hu K. T.; Zhao B. N.; Kong J. Hollow Porous Bowl-like Nitrogen-Doped Cobalt/Carbon Nanocomposites with Enhanced Electromagnetic Wave Absorption. Chem. Mater. 2021, 33, 1789–1798. 10.1021/acs.chemmater.0c04734.

[ref37] Chen Z. J.; Yang S. C.; Liu X. L.; Gao Y. H.; Dong X.; Lai X.; Zhu M. H.; Feng H. Y.; Zhu X. D.; Lu Q.; Zhao M.; Chen H. Z.; Lovell J. F.; Fang C. Nanobowl-Supported Liposomes Improve Drug Loading and Delivery. Nano Lett. 2020, 20, 4177–4187. 10.1021/acs.nanolett.0c00495. 32431154

[ref38] Wang W. H.; Jin C.; Qi L. M. Hierarchical CdS Nanorod@SnO2 Nanobowl Arrays for Efficient and Stable Photoelectrochemical Hydrogen Generation. Small 2018, 14, 1801352 10.1002/smll.201801352. 30027578

[ref39] Wei X.; Liu Z. H.; Jin X. L.; Huang L.; Gurav D. D.; Sun X. M.; Liu B. H.; Ye J.; Qian K. Plasmonic nanoshells enhanced laser desorption/ionization mass spectrometry for detection of serum metabolites. Anal. Chim. Acta 2017, 950, 147–155. 10.1016/j.aca.2016.11.017. 27916119

[ref40] Nicolardi S.; van der Burgt Y. E. M.; Codee J. D. C.; Wuhrer M.; Hokke C. H.; Chiodo F. Structural Characterization of Biofunctionalized Gold Nanoparticles by Ultrahigh-Resolution Mass Spectrometry. ACS Nano 2017, 11, 8257–8264. 10.1021/acsnano.7b03402. 28686409 PMC5616101

[ref41] Xu W.; Wang L.; Zhang R.; Sun X.; Huang L.; Su H.; Wei X.; Chen C.-C.; Lou J.; Dai H.; Qian K. Diagnosis and prognosis of myocardial infarction on a plasmonic chip. Nat. Commun. 2020, 11, 1654 10.1038/s41467-020-15487-3. 32245966 PMC7125217

[ref42] Hilvo M.; De Santiago I.; Gopalacharyulu P.; Schmitt W. D.; Budczies J.; Kuhberg M.; Dietel M.; Aittokallio T.; Markowetz F.; Denkert C.; Sehouli J.; Frezza C.; Darb-Esfahani S.; Braicu E. I. Accumulated Metabolites of Hydroxybutyric Acid Serve as Diagnostic and Prognostic Biomarkers of Ovarian High-Grade Serous Carcinomas. Cancer Res. 2016, 76, 796–804. 10.1158/0008-5472.CAN-15-2298. 26685161 PMC4762194

[ref43] Braicu E. I.; Darb-Esfahani S.; Schmitt W. D.; Koistinen K. M.; Heiskanen L.; Poho P.; Budczies J.; Kuhberg M.; Dietel M.; Frezza C.; Denkert C.; Sehouli J.; Hilvo M. High-grade ovarian serous carcinoma patients exhibit profound alterations in lipid metabolism. Oncotarget 2017, 8, 102912–102922. 10.18632/oncotarget.22076. 29262533 PMC5732699

[ref44] Paraskar A. S.; Soni S.; Chin K. T.; Chaudhuri P.; Muto K. W.; Berkowitz J.; Handlogten M. W.; Alves N. J.; Bilgicer B.; Dinulescu D. M.; Mashelkar R. A.; Sengupta S. Harnessing structure-activity relationship to engineer a cisplatin nanoparticle for enhanced antitumor efficacy. Proc. Natl. Acad. Sci. U. S. A. 2010, 107, 12435–12440. 10.1073/pnas.1007026107. 20616005 PMC2906605

[ref45] Bharti S. K.; Wildes F.; Hung C.-F.; Wu T. C.; Bhujwalla Z. M.; Penet M.-F. Metabolomic characterization of experimental ovarian cancer ascitic fluid. Metabolomics 2017, 13, 113 10.1007/s11306-017-1254-3. 29430218 PMC5804489

[ref46] Su H. Y.; Li X. X.; Huang L.; Cao J.; Zhang M. J.; Vedarethinam V.; Di W.; Hu Z. Q.; Qian K. Plasmonic Alloys Reveal a Distinct Metabolic Phenotype of Early Gastric Cancer. Adv. Mater. 2021, 33, 2007978 10.1002/adma.202007978. 33742513

[ref47] Gao Y.; Cheng F.; Fang W.; Liu X.; Wang S.; Nie W.; Chen R.; Ye S.; Zhu J.; An H.; Fan C.; Fan F.; Li C. Probing of coupling effect induced plasmonic charge accumulation for water oxidation. Natl. Sci. Rev. 2021, 8, nwaa151 10.1093/nsr/nwaa151. 34691655 PMC8288172

[ref48] Xia J. G.; Wishart D. S. Web-based inference of biological patterns, functions and pathways from metabolomic data using MetaboAnalyst. Nat. Protoc. 2011, 6, 743–760. 10.1038/nprot.2011.319. 21637195

[ref49] Li Z. Y.; Wang C.; Wang Z. Y.; Zhu C. G.; Li J.; Sha T.; Ma L. X.; Gao C.; Yang Y.; Sun Y. M.; Wang J.; Sun X. L.; Lu C. Q.; Difiglia M.; Mei Y. N.; Ding C.; Luo S. Q.; Dang Y. J.; Ding Y.; Fei Y. Y.; Lu B. X. Allele-selective lowering of mutant HTT protein by HTT-LC3 linker compounds. Nature 2019, 575, 203–209. 10.1038/s41586-019-1722-1. 31666698

[ref50] Miller H. A.; Yin X.; Smith S. A.; Hu X.; Zhang X.; Yan J.; Miller D. M.; van Berkel V. H.; Frieboes H. B. Evaluation of disease staging and chemotherapeutic response in non-small cell lung cancer from patient tumor-derived metabolomic data. Lung Cancer 2021, 156, 20–30. 10.1016/j.lungcan.2021.04.012. 33882406 PMC8138715

[ref51] Liu R. M.; Sun M.; Zhang G. W.; Lan Y. P.; Yang Z. B. Towards early monitoring of chemotherapy-induced drug resistance based on single cell metabolomics: Combining single-probe mass spectrometry with machine learning. Anal. Chim. Acta 2019, 1092, 42–48. 10.1016/j.aca.2019.09.065. 31708031 PMC6878984

[ref52] Pedersen H. K.; Gudmundsdottir V.; Nielsen H. B.; Hyotylainen T.; Nielsen T.; Jensen B. A. H.; Forslund K.; Hildebrand F.; Prifti E.; Falony G.; Le Chatelier E.; Levenez F.; Dore J.; Mattila I.; Plichta D. R.; Poho P.; Hellgren L. I.; Arumugam M.; Sunagawa S.; Vieira-Silva S.; Jorgensen T.; Holm J. B.; Trost K.; Kristiansen K.; Brix S.; Raes J.; Wang J.; Hansen T.; Bork P.; Brunak S.; Oresic M.; Ehrlich S. D.; Pedersen O.; Meta H. I. T. C. Human gut microbes impact host serum metabolome and insulin sensitivity. Nature 2016, 535, 376–381. 10.1038/nature18646. 27409811

[ref53] Wishart D. S.; Jewison T.; Guo A. C.; Wilson M.; Knox C.; Liu Y. F.; Djoumbou Y.; Mandal R.; Aziat F.; Dong E.; Bouatra S.; Sinelnikov I.; Arndt D.; Xia J. G.; Liu P.; Yallou F.; Bjorndahl T.; Perez-Pineiro R.; Eisner R.; Allen F.; Neveu V.; Greiner R.; Scalbert A. HMDB 3.0-The Human Metabolome Database in 2013. Nucleic Acids Res. 2012, 41, D801–D807. 10.1093/nar/gks1065. 23161693 PMC3531200

[ref54] Wang X. X.; Yang K. L.; Wu Q. L.; Kim L. J. Y.; Morton A. R.; Gimple R. C.; Prager B. C.; Shi Y.; Zhou W. C.; Bhargava S.; Zhu Z.; Jiang L.; Tao W. W.; Qiu Z. X.; Zhao L. J.; Zhang G. X.; Li X. Q.; Agnihotri S.; Mischel P. S.; Mack S. C.; Bao S. D.; Rich J. N. Targeting pyrimidine synthesis accentuates molecular therapy response in glioblastoma stem cells. Sci. Transl. Med. 2019, 11, eaau4972 10.1126/scitranslmed.aau4972. 31391321 PMC7568232

[ref55] Huang L.; Wang L.; Hu X. M.; Chen S.; Tao Y. W.; Su H. Y.; Yang J.; Xu W.; Vedarethinam V.; Wu S.; Liu B.; Wan X. Z.; Lou J. T.; Wang Q.; Qian K. Machine learning of serum metabolic patterns encodes early-stage lung adenocarcinoma. Nat. Commun. 2020, 11, 3556 10.1038/s41467-020-17347-6. 32678093 PMC7366718

[ref56] Xu W.; Lin J.; Gao M.; Chen Y.; Cao J.; Pu J.; Huang L.; Zhao J.; Qian K. Rapid Computer-Aided Diagnosis of Stroke by Serum Metabolic Fingerprint Based Multi-Modal Recognition. Adv. Sci. 2020, 7, 2002021 10.1002/advs.202002021. PMC7610260 33173737

[ref57] Wang W. M.; Green M.; Choi J. E.; Gijon M.; Kennedy P. D.; Johnson J. K.; Liao P.; Lang X. T.; Kryczek I.; Sell A.; Xia H. J.; Zhou J. J.; Li G. P.; Li J.; Li W.; Wei S.; Vatan L.; Zhang H. J.; Szeliga W.; Gu W.; Liu R.; Lawrence T. S.; Lamb C.; Tanno Y.; Cieslik M.; Stone E.; Georgiou G.; Chan T. A.; Chinnaiyan A.; Zou W. P. CD8(+) T cells regulate tumour ferroptosis during cancer immunotherapy. Nature 2019, 569, 270–274. 10.1038/s41586-019-1170-y. 31043744 PMC6533917

[ref58] Wang W. M.; Kryczek I.; Dostal L.; Lin H.; Tan L. J.; Zhao L. L.; Lu F. J.; Wei S.; Maj T.; Peng D. J.; He G.; Vatan L.; Szeliga W.; Kuick R.; Kotarski J.; Tarkowski R.; Dou Y. L.; Rattan R.; Munkarah A.; Liu J. R.; Zou W. P. Effector T Cells Abrogate Stroma-Mediated Chemoresistance in Ovarian Cancer. Cell 2016, 165, 1092–1105. 10.1016/j.cell.2016.04.009. 27133165 PMC4874853

[ref59] Sullivan L. B.; Luengo A.; Danai L. V.; Bush L. N.; Diehl F. F.; Hosios A. M.; Lau A. N.; Elmiligy S.; Malstrom S.; Lewis C. A.; Vander Heiden M. G. Aspartate is an endogenous metabolic limitation for tumour growth. Nat. Cell Biol. 2018, 20, 782–788. 10.1038/s41556-018-0125-0. 29941931 PMC6051729

[ref60] Numasawa K.; Hanaoka K.; Saito N.; Yamaguchi Y.; Ikeno T.; Echizen H.; Yasunaga M.; Komatsu T.; Ueno T.; Miura M.; Nagano T.; Urano Y. A Fluorescent Probe for Rapid, High-Contrast Visualization of Folate-Receptor-Expressing Tumors In Vivo. Angew. Chem., Int. Ed. 2020, 59, 6015–6020. 10.1002/anie.201914826. 31984590

[ref61] Harris H. R.; Cramer D. W.; Vitonis A. F.; DePari M.; Terry K. L. Folate, vitamin B6, vitamin B12, methionine and alcohol intake in relation to ovarian cancer risk. Int. J. Cancer 2012, 131, E518–E529. 10.1002/ijc.26455. 21953625 PMC3288483

[ref62] Chen W. L.; Wang J. H.; Zhao A. H.; Xu X.; Wang Y. H.; Chen T. L.; Li J. M.; Mi J. Q.; Zhu Y. M.; Liu Y. F.; Wang Y. Y.; Jin J.; Huang H.; Wu D. P.; Li Y.; Yan X. J.; Yan J. S.; Li J. Y.; Wang S.; Huang X. J.; Wang B. S.; Chen Z.; Chen S. J.; Jia W. A distinct glucose metabolism signature of acute myeloid leukemia with prognostic value. Blood 2014, 124, 1645–1654. 10.1182/blood-2014-02-554204. 25006128 PMC5726328

[ref63] Yuan L.; Hu Q. Y. Comparisons of three kinds of plane wave methods for the Helmholtz equation and time-harmonic Maxwell equations with complex wave numbers. J. Comput. Appl. Math. 2018, 344, 323–345. 10.1016/j.cam.2018.05.024.

[ref64] Zou H.; Hastie T. Regularization and variable selection via the elastic net. J. R. Stat. Soc. Ser. B-Stat. Methodol. 2005, 67, 301–320. 10.1111/j.1467-9868.2005.00503.x.

[ref65] Tibshirani R. Regression shrinkage and selection via the Lasso. J. R. Stat. Soc. Ser. B-Methodol. 1996, 58, 267–288. 10.1111/j.2517-6161.1996.tb02080.x.

[ref66] Butler N. A.; Denham M. C. The peculiar shrinkage properties of partial least squares regression. J. R. Stat. Soc. Ser. B-Stat. Methodol. 2000, 62, 585–593. 10.1111/1467-9868.00252.

[ref67] Quinlan J. R. Induction of decision trees. Mach. Learn. (Netherlands) 1986, 1, 81–106. 10.1007/BF00116251.

[ref68] Quirk T. J.; Quirk M.; Horton H. One-Way Analysis Of Variance (ANOVA). In Excel 2007 for Biological and Life Sciences Statistics: A Guide to Solving Practical Problems; Springer: New York, NY, 2013; pp 159–174.

