# Supplementary material for: Serum Metabolic Fingerprints on Bowl-Shaped Submicroreactor Chip for Chemotherapy Monitoring
Source: ACS Nano. 2022 Jan 31;16(2):2852–65. doi: 10.1021/acsnano.1c09864 (PMC9007521; doi:10.1021/acsnano.1c09864)
Supplement: Supplementary file 1 — nn1c09864_si_001.pdf [file nn1c09864_si_001.pdf]

Supplementary Materials for

Serum metabolic fingerprints on bowl-shaped submicroreactor chip for chemotherapy monitoring

Xia Yin,[†,#] Jing Yang,[†,‡,#] Mengji Zhang,[†,‡,#] Xinyao Wang,[ꝛ,#] Wei Xu,[†,‡] Cameron-Alexander H. Price,[¶,§] Lin Huang,[†,‡] Wanshan Liu,[†,‡] Haiyang Su,[†,‡] Wenjing Wang,[†] Hongyu Chen,[ꝛ] Guangjin Hou,[ꝛ] Mark Walker,[⊥] Ying Zhou,[‖] Zhen Shen,[‖] Jian Liu,[ꝛ,£,] Kun Qian,[†,‡,*] and Wen Di[†,*]*

[†]State Key Laboratory for Oncogenes and Related Genes, Shanghai Key Laboratory of Gynecologic Oncology, Department of Obstetrics and Gynecology, Renji Hospital, School of Medicine, Shanghai Jiao Tong University, Shanghai, 200127, P. R. China

[‡]School of Biomedical Engineering, and Med-X Research Institute, Shanghai Jiao Tong University, Shanghai, 200030, P. R. China

[ꝛ]State Key Laboratory of Catalysis, Dalian Institute of Chemical Physics, Chinese Academy of Sciences, Dalian, Liaoning, 116023, P. R. China

[¶]The University of Manchester at Harwell, Diamond Light Source, Didcot, Oxfordshire, OX11 0DE, UK

[§]UK Catalysis Hub, Research Complex at Harwell, Rutherford Appleton Labs, Harwell Campus, Didcot, Oxfordshire, OX11 0FA, UK

[⊥]Department of Obstetrics and Gynecology, University of Ottawa, Ottawa, Ontario, ON K1H 8L6, Canada

[‖]Department of Obstetrics and Gynecology, The First Affiliated Hospital of USTC, Division of Life Sciences and Medicine, University of Science and Technology of China, Hefei, Auhui, 230001, P. R. China

[£]DICP-Surrey Joint Centre for Future Materials, Department of Chemical and Process Engineering, and Advanced Technology Institute, University of Surrey, Guilford, Surrey, GU2 7XH, UK

*Corresponding author. Email: jian.liu@surrey.ac.uk; k.qian@sjtu.edu.cn; diwen@renji.com

# These authors contributed equally to this work.

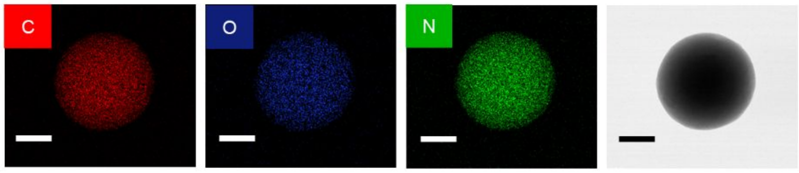

Supporting Figure 1. The elemental mapping characterization of APF-sphere. The elements of C, O, and N are indicated by red, blue, and green. The scale bar is 100 nm.

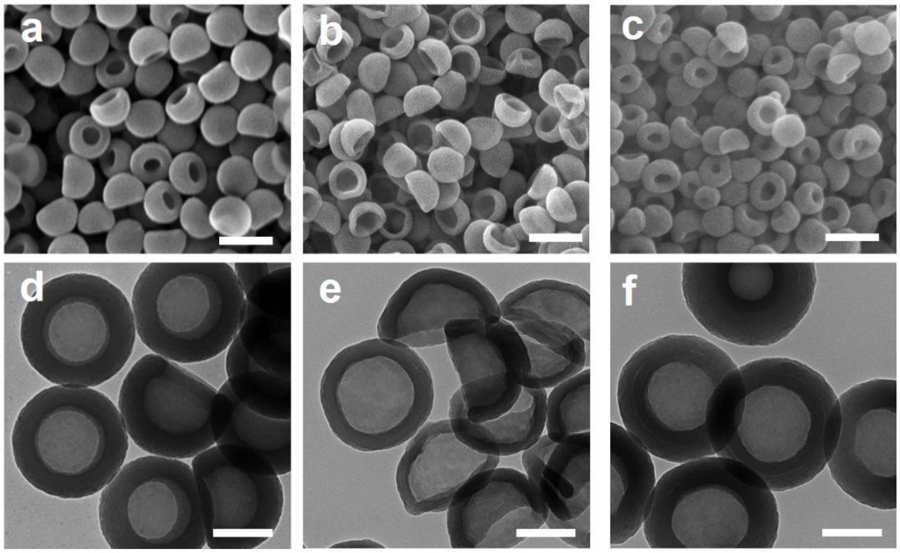

Supporting Figure 2. The characterization of APF-bowl obtained by solvent-assisted repolymerization process for 5 min (a, d), 20 min (b, e), and 180 min (c, f). The scanning electron microscopy (SEM) images (a-c, Scale bar, 500 nm) and transmission electron microscopy (TEM) images (d-f, Scale bar, 200 nm), respectively.

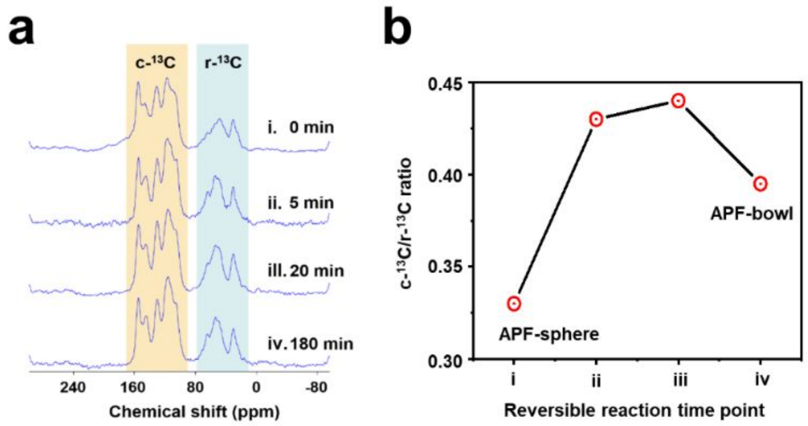

Supporting Figure 3. The characterization of ^{13}C nuclear magnetic resonance (^{13}C NMR). (a) ^{13}C CP/MAS NMR spectra of APF-sphere (0 min) and APF-bowl obtained after the reaction time of 5 min, 20 min, and 180 min. The c-^{13}C and r-^{13}C refer to the carbon content ratio on the branched-chain and benzene ring, respectively. (b) Trend diagram of c-13C/r-13C ratio of samples in reversible de/re-polymerization process, which reaction time was (i) 0 min, (ii) 5 min, (iii) 20 min, and (iv) 180 min.

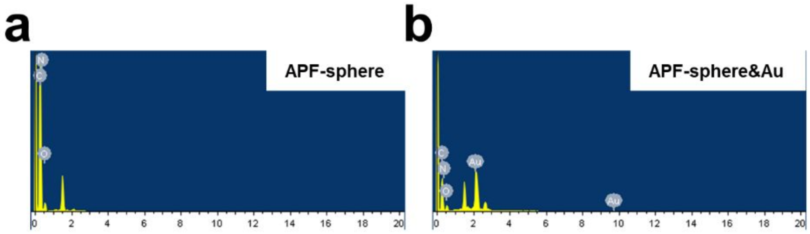

c

Materials	Weight (%)				Atomic (%)			
	C	N	O	Au	C	N	O	Au
APF-sphere	70.4	13.83	15.77		74.82	12.6	12.58	
APF-sphere&Au	37.41	6.73	8.71	47.15	71.13	10.97	12.43	5.47

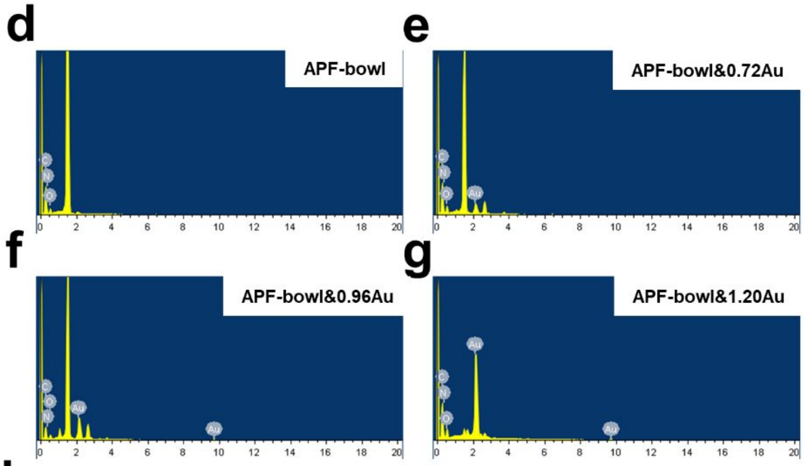

h

Materials	Weight (%)				Atomic (%)			
	C	N	O	Au	C	N	O	Au
APF-bowl	48.29	21.9	29.81		53.98	20.99	25.02	
APF-bowl&0.72Au	45.17	14.08	23.93	16.82	59.25	15.84	23.57	1.35
APF-bowl&0.96Au	30.05	8.86	14.33	46.76	58.62	14.82	20.99	5.56
APF-bowl&1.20Au	26.26	6.57	8.20	58.98	63.06	13.52	14.78	8.64

Supporting Figure 4. The energy dispersive X-ray spectra (EDS) characterization of sphere-shaped and bowl-shaped chips. The EDS of APF-sphere and APF-sphere&Au are displayed in (a) and (b), respectively. The elemental contents of C, N, O, and Au are summarized in Supporting Figure 4c. The EDS of APF-bowl, APF-bowl&0.72Au, APF-bowl&0.96Au, and APF-bowl&1.20Au, are displayed in (d), (e), (f) and (g), respectively. The elemental contents of C, N, O, and Au are summarized in Supporting Figure 4h.

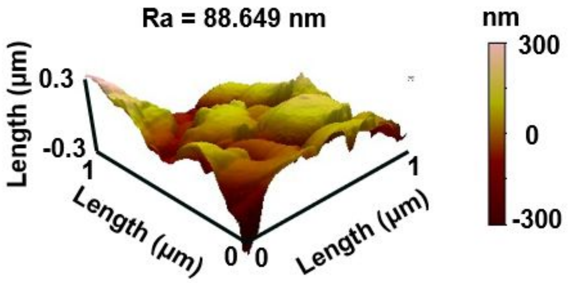

Supporting Figure 5. The 3D plot of atomic force microscopy (AFM) image for the characterization of the APF-bowl&0.96Au surface roughness.

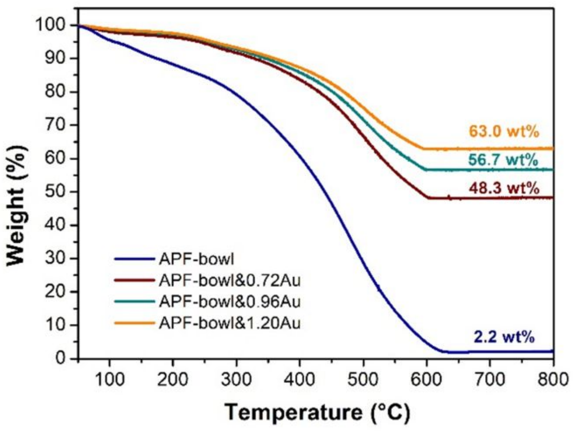

Supporting Figure 6. The thermogravimetric (TG) characterizations of APF-bowl and APF-bowl&*M*Au (*M* = 0.72, 0.96, 1.20).

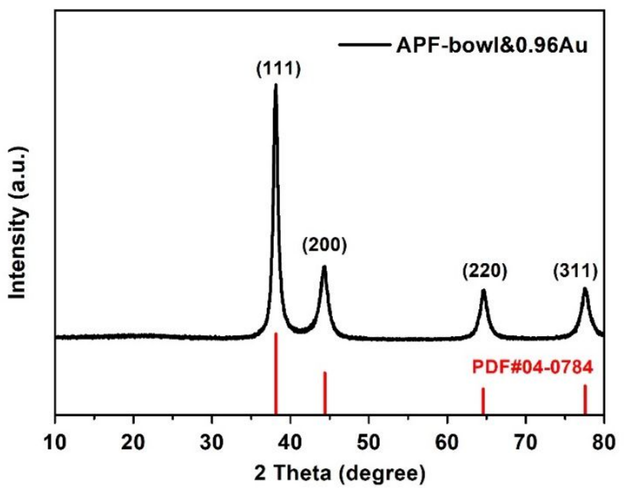

Supporting Figure 7. The X-ray Diffraction (XRD) pattern of APF-bowl&0.96Au. The typical peaks at 2θ of 38.2°, 44.4°, 64.6°, and 77.5° were assigned to rings of (111), (200), (220) and (311) of Au.

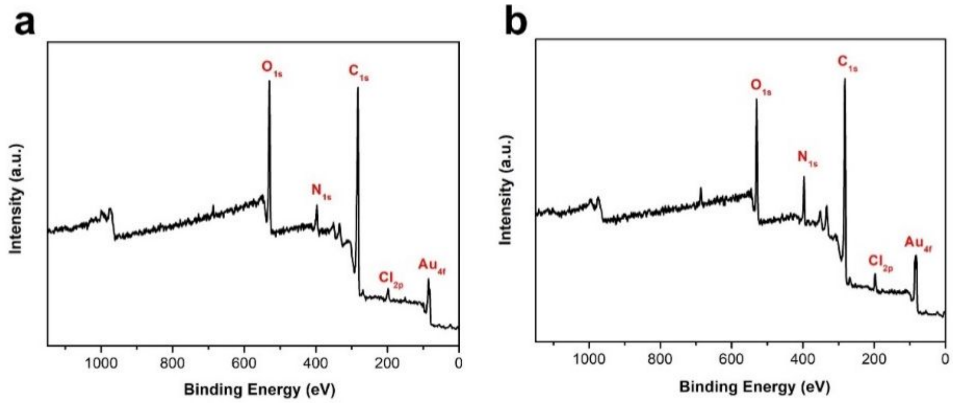

Supporting Figure 8. The X-ray photoelectron spectroscopy (XPS) characterization. The full spectra of (a) APF-sphere&Au and (b) APF-bowl&Au, are displayed, respectively.

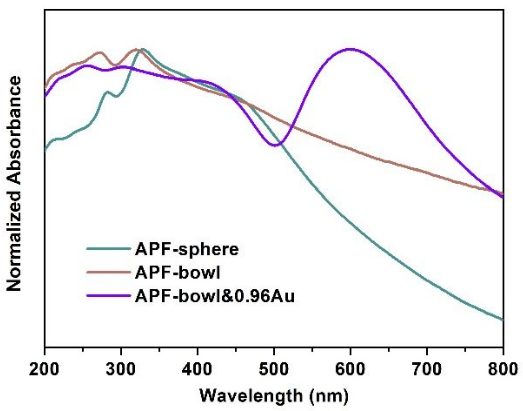

Supporting Figure 9. Ultraviolet-visible (UV-vis) absorbance spectra of APF-sphere, APF-bowl, and APF-bowl&0.96Au.

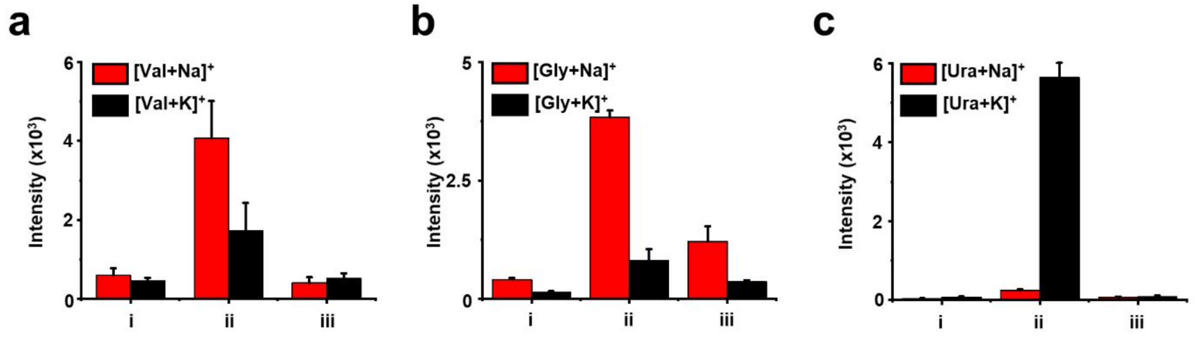

Supporting Figure 10. The bar graph of laser desorption/ionization mass spectrometry (LDI MS) detection. Mean signal intensities of sodium and potassium adducted ($[M+Na]^+$and $[M+K]^+$) signals of (a) valine, (b) glycyl-glycine, and (c) uracil, by using the matrix of (i) APF-sphere&0.72Au, (ii) APF-sphere&0.96Au, and (iii) APF-sphere&1.20Au. Three independent LDI MS experiments were conducted, and the small metabolites were at a concentration of 1 ng/nL.

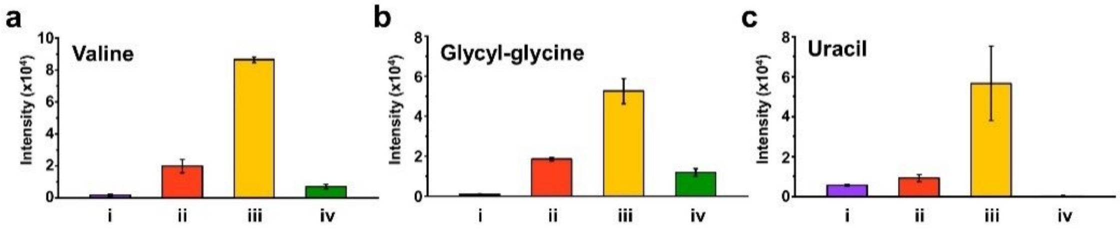

Supporting Figure 11. The bar graph of LDI MS detection. Mean signal intensities of potassium adducted ($[M+K]^+$) signals of (a) valine, (b) glycyl-glycine, and (c) uracil, by using the matrix of (i) APF-sphere&Au, (ii)APF-bowl&0.72Au, (iii) APF-bowl&0.96Au, and (iv) APF-bowl&1.20Au. Three independent LDI MS experiments were conducted, and the small metabolites were at a concentration of 1 ng/nL.

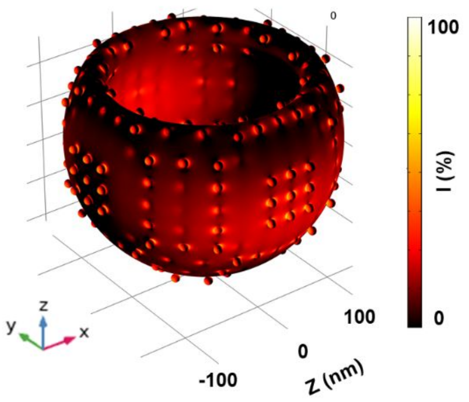

Supporting Figure 12. The uniform distribution of Au nanoparticles was adopted for calculating the space of neighboring Au nanoparticles in APF-bowl&0.96Au, which was 25.83 nm.

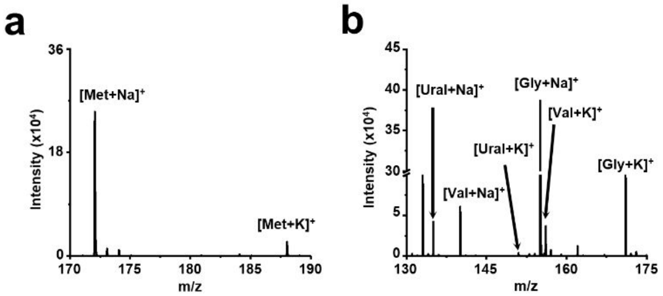

Supporting Figure 13. The protein tolerance of APF-bowl&0.96Au in LDI MS detection. (a) The mass spectrum of the mixture of methionine (Met, 1 ng/nL) and BSA (5mg/mL). **(b)** The mass spectrum of the mixture of BSA (5mg/mL) and typical small metabolites (including valine (Val), glycyl-glycine (Gly), and uracil (Ura), each at a concentration of 1 ng/nL). The sodium adducts ($[M+Na]^+$) and potassium adducts ($[M+K]^+$) of small metabolites were labeled in the figure.

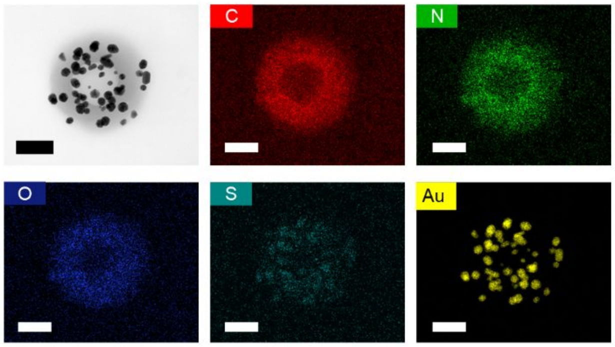

Supporting Figure 14. The elemental mapping characterizations. Elemental mappings of C, N, O, Au, and S for a submicroreactor-methionine mixture. The scale bars were 100 nm.

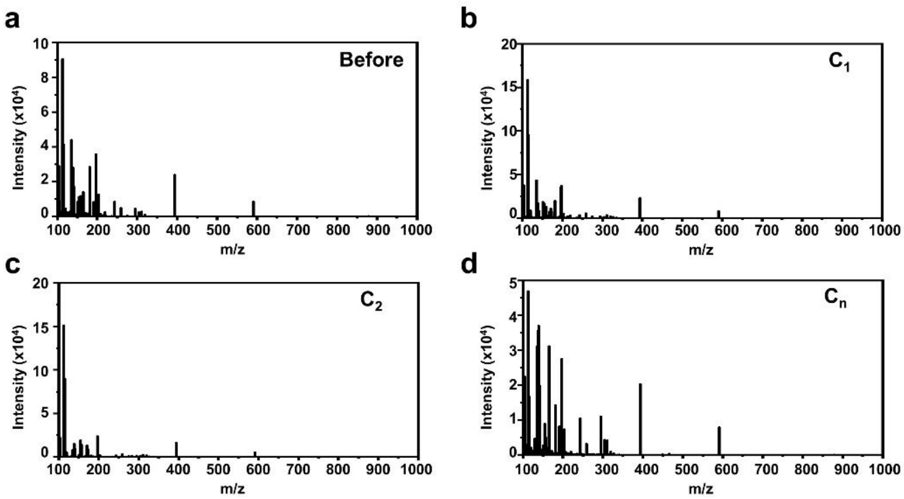

Supporting Figure 15. The typical MS spectra of ovarian cancer patients before and after chemotherapy. The MS spectrum of an ovarian cancer patient (a) before chemotherapy, (b) after cycle 1 chemotherapy (C_1), (c) after cycle 2 chemotherapy (C_2), and (d) after cycle *n* chemotherapy (C_n, $n \geq 5$), by using APF-bowl&0.96Au as the matrix. The LDI MS experiments were all conducted in the positive ion mode, and the matrix suspension of APF-bowl&0.96Au was 1 ng/nL.

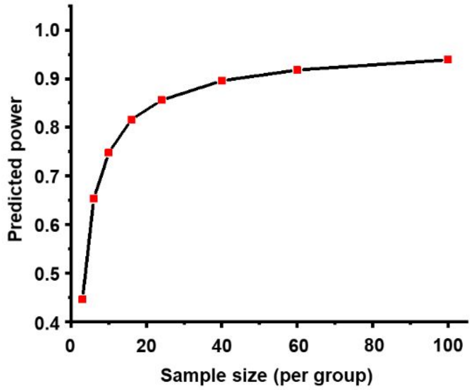

Supporting Figure 16. Power analysis based on a pilot dataset. The 10 SMFs (5/5, before chemotherapy/after chemotherapy) were analyzed to obtain the minimum required sample for a robust machine learning model. The minimum sample size of 16 per group will achieve a predicted power of ~0.8.

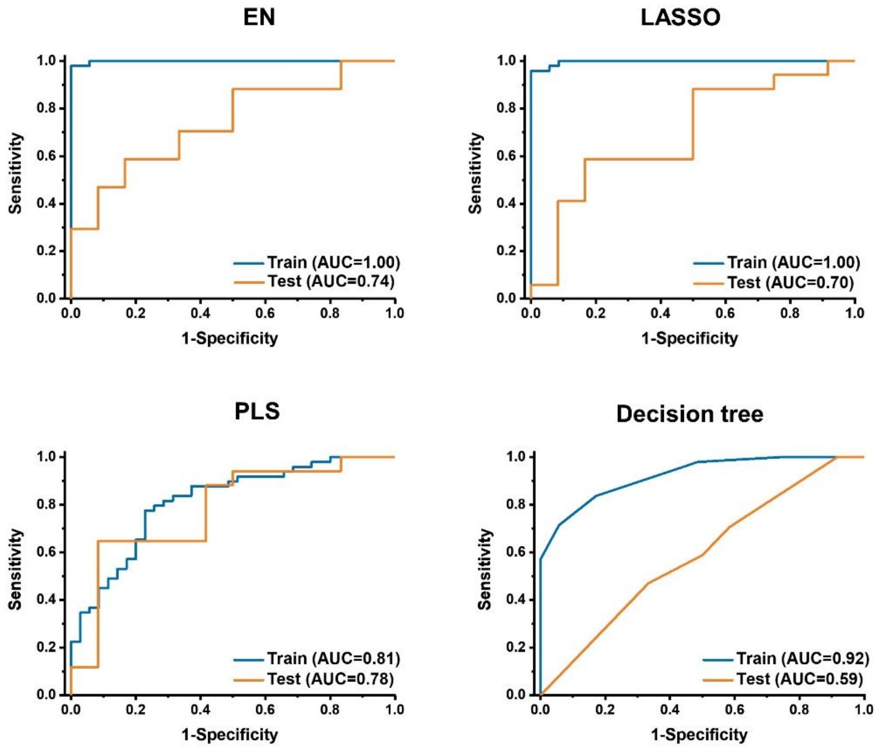

Supporting Figure 17. Machine learning of serum metabolic fingerprints (SMFs) for differentiation between the ovarian cancer patients before chemotherapy and in C_1 group. The machine learning methods of (a) elastic net (EN), (b) the least absolute shrinkage and selection operator (LASSO), (c) partial least squares (PLS), and (d) decision tree, were respectively applied. The PLS was demonstrated as the best candidate for differentiation of patients before chemotherapy and patients in C_1 group. The receiver operating characteristics (ROC) curves of training and test set are respectively presented in blue and yellow with area-under-the-curve (AUC) labeled.

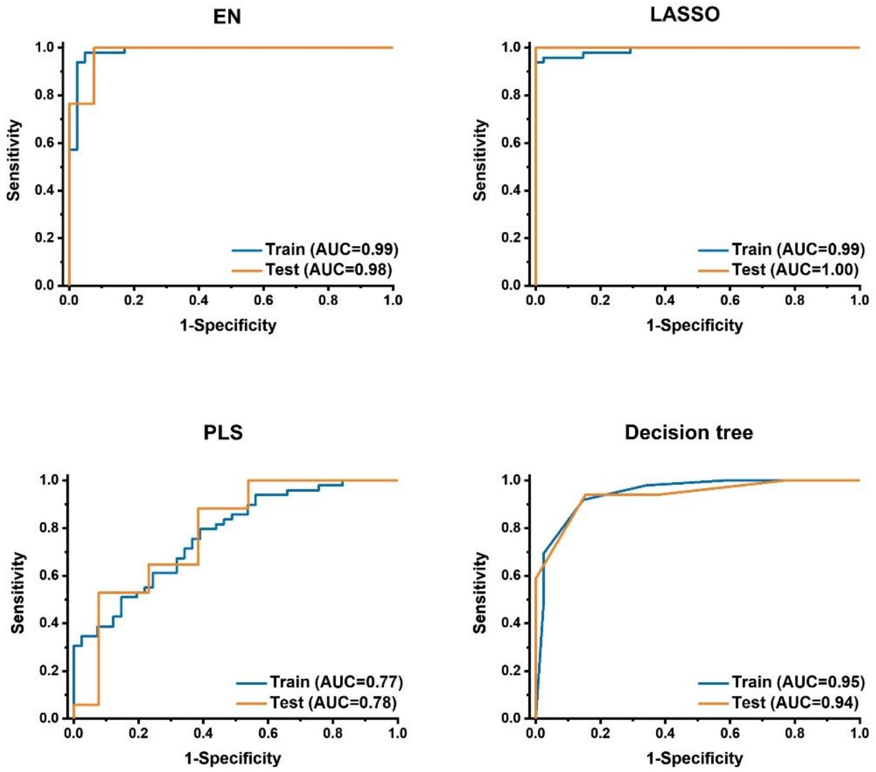

Supporting Figure 18. Machine learning of SMFs for differentiation between the ovarian cancer patients before chemotherapy and in C_2 group. The machine learning method of (a) EN, (b) LASSO, (c) PLS, and (d) decision tree, were respectively applied. LASSO was demonstrated as the best candidate for differentiation of patients before chemotherapy and in C_2 group. The ROC curves of training and test set are respectively presented in blue and yellow with AUC labeled.

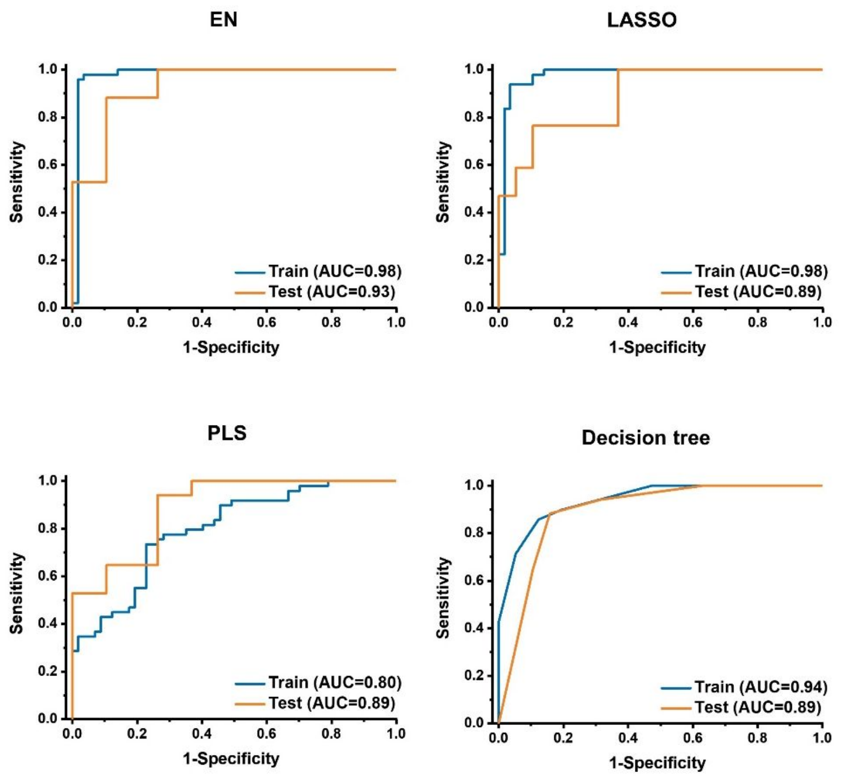

Supporting Figure 19. Machine learning of SMFs for differentiation between the ovarian cancer patients before chemotherapy and in C_n group. The machine learning methods of (a) EN, (b) LASSO, (c) PLS, and (d) decision tree, were respectively applied. LASSO was demonstrated as the best candidate for differentiation of patients before chemotherapy and in C_n group. The ROC curves of training and test set are respectively presented in blue and yellow with AUC labeled.

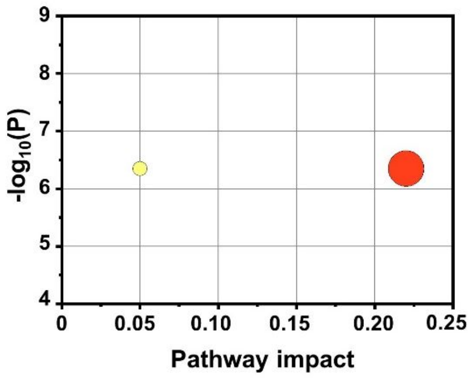

Supporting Figure 20. The pathway analysis based on the established biomarker panel for chemotherapy monitoring. Two pathways were identified to correlate with the established metabolic biomarker panel (Supporting Table 6), in which the glyoxylate and dicarboxylate metabolism with pathway impact of 0.22 are presented in the red circle and (2) Glycine, serine, and threonine metabolism with pathway impact of 0.05 were presented in the yellow circle. The size of the circle correlates with corresponding pathway impact.

Supporting Table 1. The diverse biomedical applications by regulating surface roughness.

No.	Reference	Materials with surface roughness	Property	Biomedical Application
This study	—	APF-bowl&0.96Au	Increased adsorption site and high selectivity for metabolites, and plasmon transfer within the nano-scaled cavities to facilitate LDI efficiency	Submicroreactor for metabolite detection
**Ref (1)**	Wang *et al*. *Adv. Healthcare Mater*. **2018**, 7, 1800318	The mixture of Mg particles and gallium-indium alloy	Excellent thermal conductivity, favorable formability, and benign biocompatibility	Photothermal therapy
**Ref (2)**	Chen *et al*. *ACS Nano* **2018**, 12, 6, 5646–5656	Rattle-structured rough nanocapsules (Au@HSN-PGEA, AHPs)	Desirable surface roughness and NIR responsiveness	
**Ref (3)**	Song *et al*. *ACS Nano* **2017**, 11, 6, 6102-6113	Branched nanoporous gold nanoshells	Enhanced physico-optical properties for laser irradiation triggered drug release	Drug/cellular delivery
**Ref (4)**	Niu *et al*. *Adv. Mater.* **2013**, 25, 43, 6233-6237	Nanoparticles mimicking virus surface topography	Enhanced biomolecules binding and cellular uptake efficacy	
**Ref (5)**	Kocer *et al*. *Adv. Mater*. **2017**, 29. 27, 1606407	Light responsive liquid crystal polymer networks	Switched cell migration patterns upon in situ temporal changes in surface nano-roughness	Cell adhesion, migration, and regulation
**Ref (6)**	Zhou *et al*. ***J. Mater. Chem. B*** **2015**, **3**, 4439-4450	Ultrafine poly(L-lactic acid) (PLLA) microfibers	Anisotropic wettability and greater protein adsorption	
**Ref (7)**	Li *et al*. *ACS Appl. Mater. Interfaces* **2021**, 13, 27, 32205–32216	Stimuli-responsive lysozyme nanocapsules (NCP)	Remarkably reduced adsorption of proteins, polysaccharides, and bacteria	Antibacterial
**Ref (8)**	Xu *et al*. *J. Hazard Mater.* **2018**, 5, 343, 285-297	PET fabrics coated with TiO2nanowires	Excellent UV photocatalytic activity	
**Ref (9)**	Duy *et al*. *Nanoscale*, **2019**, 11, 16455-16462	Si nanopillar array	Against Gram-negative Pseudomonas and Staphylococcus aureus due to nanostructure-induced rupture.	
**Ref (10)**	Shi *et al*. *Small* **2018**, 14, 27, e1800819	Lotus leaf onto PDMS substrates	Increased contact area and high sensitivity as a flexible piezoresistive pressure sensor	Sensors
**Ref (11)**	Qi *et al*. *Sens. Actuators B Chem*. **2019**, 279, 170-176	3D sulfur/nitrogen co-doped graphene	Detection of catechol and hydroquinone with low detection limits	
**Ref**	Shu *et al*. S*mall*	Au nanoparticles	Fast, sensitive, and selective	

**(12)**	*Methods* **2020**, 4, 4, 1900469	deposited on a dopamine-bubble layer	detection of small metabolites	

Supporting Table 2. Comparison between the present APF-bowl&Au with previous reported anisotropic morphologies as LDI MS matrix.

No.	Reference	Matrix	Substrate	Property for LDI MS detection	Detected molecules	Application
This study	—	APF-bowl&Au	APF-bowl	Enhanced laser desorption/ionization efficiency and desirable adsorption of small molecule metabolites	Serum metabolic fingerprints (MW < 1000 Da)	Chemotherapy monitoring
**Ref (13)**	Tseng *et al. Anal. Chem.* **2018,** *90* (12), 7283-7291	Self-Assembled Chiral Gold Supramolecules	—	High absorption efficiency of laser energy, analyte-binding capacity, and homogeneity	Carnitine (MW > 1000 Da)	Targeted molecule detection
**Ref (14)**	Yoon *et al. Chem. Comm.* **2018,** *54* (45), 5688-5691	Donut-shaped C18-Au surface	Au surface	Simplified sample preparation process	Neuropeptide (MW > 1000 Da)	
**Ref (15)**	Li *et al. Chem. Comm.* **2019,** *55* (15), 2166-2169	Au nanobowls	Au nanobowls	High stability with reduced analysis time	Oligonucleotides (MW > 1000 Da)	
**Ref (16)**	Ge *et al. J. Chromatogr. A.* **2020,** *1615*.	Apatamer-Au doped COFs	COFs	Selective and satisfactory extraction property to insulin	Insulin (MW > 1000 Da)	
**Ref (17)**	Huang *et al. Anal. Chem.* **2018,** *90* (14), 8607-8615.	Au/diphenylalanine nanosheets	Diphenylalanine nanosheets	Laser light absorption ability	Anabolic steroids and estrogens (MW < 1000 Da)	
**Ref (18)**	Wang *et al. ACS Appl. Mater. Interfaces* **2020,** *12* (38), 42567-42575.	Porous TiO_2 film immobilized with gold nanoparticles (AuNPs-FPTDF)	Porous TiO_2 film	High detection sensitivity, good repeatability, and low background noise	Various small molecules (*e.g.,* amino acids) (MW < 1000 Da)	
**Ref (19)**	Kim *et al. Small* **2021,** *17* (49), 2103745	Au nanoisland functionalized ZnO nanotubes (AuNI-ZNTs).	ZnO nanotubes	Favorable desorption process	Fatty acids and monosaccharides (MW < 1000 Da)	
**Ref (20)**	Kim *et al. Adv. Funct. Mater.* **2021,** *31* (29), 2102475.	Au-modified TiO_2 nanowires (npAu-TNW)	TiO_2 nanowires	Efficient photo thermal conversion	Neurotransmitter (MW < 1000 Da)	
**Ref (21)**	Kim *et al. ACS Appl. Mater. Interfaces* **2019,** *11* (22), 20509-20520	Au nanoislands/TiO_2 nanowires	TiO_2 nanowires	Improved desorption and ionization performance of the heterostructure	Immunosuppressors (MW < 1000 Da)	

**Ref (22)**	Li *et al*. *J. Hazard. Mater*. **2022,** *423*, 126893	AuNPs/NiFe-LDHs	NiFe-LDHs	High peak intensity and low background noise	Metronidazole (MW < 1000 Da)	
**Ref (23)**	Alexandra *et al. ACS Nano* **2020,** *14* (6), 6785-6794	Au-coated black silicon substrates	Black silicon substrates	Efficient transference of metabolites from the tissues to the substrate surface	Animal tissues and human fingerprints	Untargeted molecule detection

Supporting Table 3. The intensity of typical small molecule metabolites by LDI MS detection.

Analyte [1]	Matrix [2]	[M+Na]+		[M+K]+	
		Intensity [3]	S/N[4]	Intensity [3]	S/N[4]
Valine	APF-bowl	98.24±9.91	<3	71.33±18.58	<3
	APF-bowl&0.72Au	25009.70±706.00	38.33±8.08	19847.00±4105.37	22.67±2.08
	APF-bowl&0.96Au	84296.30±3165.98	452.33±35.73	86458.70±1740.00	368±54.81
	APF-bowl&1.20Au	12798.00±605.31	197.00±25.94	7022.33±1412.69	90.00±8.54
	APF-sphere&Au	4075.33±936.84	105.00±19.05	1724.00±705.82	42.33±16.92
Glucose	APF-bowl	14.33±4.04	<3	8.67±4.62	<3
	APF-bowl&0.72Au	22110.43±2226.08	13.17±3.65	6928.56±2044.43	<3
	APF-bowl&0.96Au	31812.11±628.43	22.97±6.96	7527.68±1153.59	<3
	APF-bowl&1.20Au	11596.84±710.45	4.77±1.46	7020.84±1096.67	<3
	APF-sphere&Au	311.00±52.68	4	369.33±79.22	4.67±0.58
Decanoic acid	APF-bowl	2.67±1.15	<3	2.00±2.00	<3
	APF-bowl&0.72Au	2554.67±211.68	<3	6383.89±2327.44	13.67±3.43
	APF-bowl&0.96Au	4873.83±650.27	6.53±0.93	23072.09±2143.88	28.20±2.31
	APF-bowl&1.20Au	1389.84±236.56	7.95±0.21	7120.56±1938.47	40.57±8.00
	APF-sphere&Au	25.00±10.15	<3	454.60±132.26	39.40±11.78
Glycyl-glycine	APF-bowl	8.33±6.03	<3	3.67±4.62	<3
	APF-bowl&0.72Au	79122.19±703.42	552.83±72.79	18469.92±854.70	103.90±13.57
	APF-bowl&0.96Au	102523.00±3693.55	895.37±122.61	52491.24±6294.13	353.77±59.98
	APF-bowl&1.20Au	47228.93±2711.96	423.83±99.95	11814.52±1894.13	83.53±25.27
	APF-sphere&Au	3840.57±136.77	121.07±23.85	805.29±240.54	22.80±1.92
Uracil	APF-bowl	10.33±3.51	<3	1.67±1.53	<3
	APF-bowl&0.72Au	1648.97±471.5	16.10±5.52	9156.38±1776.74	65.30±13.40
	APF-bowl&0.96Au	10079.41±2383.43	127.73±61.05	56563.98±18589.20	550.30±301.43
	APF-bowl&1.20Au	395.67±43.94	<3	274.2±17.36	9.30±0.14
	APF-sphere&Au	242.09±33.81	6.80±1.90	5636.52±370.03	132.90±40.07
Leucine	APF-bowl	9±2.65	<3	10.00±4.36	<3
	APF-bowl&0.72Au	1739.34±636.98	159.17±60.71	836.9±419.77	74.80±36.63
	APF-bowl&0.96Au	2738.58±1729.88	227.4±146.09	7056.78±2583.48	592.20±223.28
	APF-bowl&1.20Au	1369.22±282.99	121.07±23.23	533.95±150.17	46.43±13.06
	APF-sphere&Au	45.08±19	4.2±1.9	175.62±32.97	16.40±2.46

[1] Six typical metabolites in human serum, including valine, glucose, decanoic acid, glycyl-glycine, uracil, and leucine, were utilized for evaluating the detection performance of different matrices in LDI MS.

[2] The matrices, including APF-bowl, APF-bowl&0.72Au, APF-bowl&0.96Au, APF-bowl&1.20Au, and APF-sphere&Au, were applied for LDI MS detection with the concentration of 1 ng/nL.

[3] The intensity of sodium and potassium adducts of small molecule metabolites ($[M+Na]^+$ and $[M+K]^+$) was calculated by three independent experiments with standard deviation.
[4] S/N refers to the signal to noise ratio of sodium and potassium adducts of small molecule metabolites, which was calculated by three independent experiments with standard deviation.

Supporting Table 4.The total ion count (TIC) of mass spectra from a representative serum sample of an ovarian cancer patient.

Matrix	TIC			
	1	2	3	Average [1]
APF-sphere&Au	739900	710963	643666	698177±49375
APF-bowl&0.72Au	1443224	1350514	1342195	1378644±56082
APF-bowl&0.96Au	5889989	6108490	6594293	6197590±360507
APF-bowl&1.20Au	956918	955830	976908	963219±11868

[1] The averaged TIC with standard deviation was calculated based on three independent experiments.

Supporting Table 5. Demographic information and clinical feature of ovarian cancer patients.

Patient ID	Age	Physiological type [1]	Disease stage [2]	Serum sample available [3]			
				Before	C_1	C_2	C_n
**1**	57	MOC	IIC	√			√
**2**	60	HGSOC	II	√	√		√
**3**	49	HGSOC	IV	√	√	√	
**4**	57	HGSOC	IA	√		√	
**5**	52	HGSOC	III	√	√	√	
**6**	52	HGSOC	IV	√		√	
**7**	70	HGSOC	IIIC	√	√		
**8**	61	HGSOC	IIIC	√	√	√	
**9**	56	HGSOC	IIIC	√	√		
**10**	56	HGSOC	IIIC	√		√	√
**11**	62	HGSOC	IIIC	√			√
**12**	75	HGSOC	IIIC	√	√		
**13**	63	HGSOC	IC	√	√		√
**14**	51	HGSOC	IC	√	√	√	
**15**	65	EOC	IA	√		√	
**16**	54	HGSOC	IA	√			√
**17**	67	HGSOC	IV	√	√		√
**18**	65	HGSOC	IIB	√		√	
**19**	74	OCCC	IIIC	√	√	√	√
**20**	61	HGSOC	IIB	√		√	√
**21**	61	HGSOC	IIIC	√	√	√	
**22**	46	HGSOC	IIIC	√	√		√
**23**	56	HGSOC	IIIC	√	√	√	
**24**	64	HGSOC	IIIC	√	√	√	√
**25**	48	HGSOC	IIA	√	√	√	
**26**	50	HGSOC	IC	√	√	√	
**27**	60	HGSOC	IC	√	√	√	√
**28**	60	HGSOC	IIB	√	√	√	
**29**	64	HGSOC	IA	√	√	√	√
**30**	65	HGSOC	IIIC	√		√	√
**31**	49	HGSOC	IIIC	√	√	√	
**32**	63	HGSOC	IV	√	√	√	√
**33**	80	HGSOC	IIIC	√	√	√	
**34**	36	HGSOC	IC	√		√	
**35**	37	OCCC	IIIC	√	√	√	√
**36**	71	LGSOC	III	√	√	√	√
**37**	30	HGSOC	IIIC	√	√	√	√
**38**	71	MOC	IC	√	√	√	
**39**	36	OCCC	IV	√		√	
**40**	66	HGSOC	IIIC	√	√	√	√
**41**	41	HGSOC	IIIA	√			√

**42**	65	HGSOC	IIIC	√			
**43**	53	HGSOC	IV	√		√	√
**44**	63	HGSOC	IIIC	√	√		
**45**	57	HGSOC	IV	√	√	√	
**46**	38	HGSOC	III	√	√	√	√
**47**	61	HGSOC	III	√	√	√	√
**48**	73	HGSOC	IIIC	√	√	√	√
**49**	48	HGSOC	IIIC	√	√	√	√
**50**	35	HGSOC	IIB	√	√	√	√
**51**	64	HGSOC	IIIC	√	√	√	√
**52**	66	HGSOC	IV		√	√	√
**53**	53	HGSOC	IIIC	√	√	√	√
**54**	59	HGSOC	IIIC	√		√	√
**55**	44	HGSOC	IIIC	√	√	√	√
**56**	64	HGSOC	IIIC	√	√	√	√
**57**	66	LGSOC	IV	√	√	√	√
**58**	63	HGSOC	IIIC	√	√	√	√
**59**	54	HGSOC	IIB	√	√		√
**60**	50	HGSOC	IIIC	√		√	√
**61**	44	HGSOC	IIIC	√		√	√
**62**	65	HGSOC	IV	√		√	√
**63**	56	HGSOC	IIIC	√		√	√
**64**	53	HGSOC	IIIC	√	√	√	√
**65**	64	HGSOC	IIIC	√	√	√	√
**66**	51	HGSOC	IIIC	√	√	√	√
**67**	62	OCCC	I	√	√	√	√
**68**	53	OCCC	IIIC			√	√

[1] The physiological type was obtained from the pathological examination, including 58 cases of high-grade serous ovarian carcinoma (HGSOC), 5 cases of ovarian clear cell carcinoma (OCCC), 2 cases of mucinous ovarian carcinoma (MOC), 1 case of endometrioid ovarian carcinoma (EOC), and 2 cases of low grade serous ovarian carcinoma (LGSOC).

[2] The disease stage was obtained according to the standards of Federation International of Gynecology and Obstetrics (FIGO) 2018 for ovarian cancer.

[3] The serum sample available for the patient before chemotherapy (Before), after cycle 1 of chemotherapy (C_1), C_2, and C_n ($n \geq 5$) is marked with '√'.

Supporting Table 6. The diagnostic parameters of before chemotherapy and chemotherapy cycle 1 (C_1) by elastic net (EN), least absolute shrinkage and selection operator (LASSO), partial least squares (PLS) regression, and decision tree.

Machine learning methods	Diagnostic performance	Training set (before/C_1, 49/35)	Test set (before/C_1, 17/12)
**EN**	AUC	1.00	0.74
	Sensitivity (%)	0.98	0.59
	Specificity (%)	1.00	0.83
	Accuracy (%)	0.98	0.66
**LASSO**	AUC	1.00	0.70
	Sensitivity (%)	0.96	0.59
	Specificity (%)	1.00	0.83
	Accuracy (%)	0.96	0.66
**PLS**	AUC	0.81	0.78
	Sensitivity (%)	0.78	0.65
	Specificity (%)	0.77	0.92
	Accuracy (%)	0.76	0.72
**Decision tree**	AUC	0.92	0.59
	Sensitivity (%)	0.84	0.47
	Specificity (%)	0.83	0.67
	Accuracy (%)	0.81	0.55

Supporting Table 7. The diagnostic parameters of before chemotherapy and chemotherapy cycle 2 (C_2) by EN, LASSO, PLS, and decision tree.

Machine learning method	Diagnostic performance	Training set (before/C_2, 49/41)	Test set (before/C_2, 17/13)
**EN**	AUC	0.99	0.98
	Sensitivity (%)	0.96	0.83
	Specificity (%)	0.98	1.00
	Accuracy (%)	0.95	0.92
**LASSO**	AUC	0.99	1.00
	Sensitivity (%)	0.96	0.93
	Specificity (%)	0.96	1.00
	Accuracy (%)	0.98	1.00
**PLS**	AUC	0.77	0.78
	Sensitivity (%)	0.69	0.70
	Specificity (%)	0.76	0.88
	Accuracy (%)	0.63	0.62
**Decision tree**	AUC	0.95	0.94
	Sensitivity (%)	0.82	0.90
	Specificity (%)	0.92	0.94
	Accuracy (%)	0.85	0.85

Supporting Table 8. The diagnostic parameters of before chemotherapy and chemotherapy cycle *n* (C_n) based on EN, LASSO, PLS, and decision tree.

Machine learning method	Diagnostic performance	Training set (before/C_n, 49/57)	Test set (before/C_n, 17/19)
**EN**	AUC	0.98	0.93
	Sensitivity (%)	0.98	0.88
	Specificity (%)	0.96	0.89
	Accuracy (%)	0.96	0.86
**LASSO**	AUC	0.98	0.89
	Sensitivity (%)	0.94	0.76
	Specificity (%)	0.96	0.89
	Accuracy (%)	0.94	0.81
**PLS**	AUC	0.80	0.89
	Sensitivity (%)	0.73	0.94
	Specificity (%)	0.77	0.74
	Accuracy (%)	0.75	0.75
**Decision tree**	AUC	0.94	0.89
	Sensitivity (%)	0.86	0.88
	Specificity (%)	0.88	0.84
	Accuracy (%)	0.84	0.86

Supporting Table 9. The potential biomarkers selected from SMFs.

No.	Metabolite [1]	HMDB ID [2]	m/z	Adduct	p_1 [3]	p_2 [4]	p_3 [5]
**1**	Hydroxybutyric acid	HMDB0001352	103.95	$[M]^+$	**	**	****
**2**	Maleic acid	HMDB0000176	116.84	$[M+H]^+$	*	**	****
**3**	D-Cysteine	HMDB0003417	121.96	$[M+H]^+$	***	****	****
**4**	N-Acetylasparagine	HMDB0006028	212.95	$[M+K]^+$	**	**	****
**5**	3-Hydroxy-2-methylpyridine-4,5-dicarboxylate	HMDB0006955	235.95	$[M+K]^+$	**	***	****
**6**	Dihydroneopterin phosphate	HMDB0006824	335.85	$[M+H]^+$	*	***	****

[1] The metabolites include hydroxybutyric acid, maleic acid, D-cysteine, N-acetylasparagine, 3-hydroxy-2-methylpyridine-4,5-dicarboxylate, and dihydroneopterin phosphate, which were screened out by machine learning methods and constructed as the metabolic biomarker panel for chemotherapy monitoring.

[2] refers to the ID of the corresponding metabolite in the human metabolite database (https://hmdb.ca/).

[3] The p_1 value was calculated by the t-test based on the intensities between ovarian cancer patients before chemotherapy and after cycle 1 chemotherapy (C_1) at the corresponding m/z value.

[4] The p_2 value was calculated by the t-test based on the intensities between ovarian cancer patients before chemotherapy and after cycle 2 chemotherapy (C_2) at the corresponding m/z value.

[5] The p_3 value was calculated by the t-test based on the intensities between ovarian cancer patients before chemotherapy and after cycle *n* chemotherapy (C_n, $n \geq 5$) at corresponding m/z value. (* represented $p < 0.05$, ** represented $p < 0.01$, *** represented $p < 0.001$, and **** represented $p < 0.0001$).

Supporting Table 10. The statistics of potential biomarkers after variable cycle of chemotherapy.

No.	Metabolite	p (C_1 vs C_n)	p (C_2 vs C_n)	Fold change [1]	Log_2(Fold change) [1]
**1**	Hydroxybutyric acid	*	*	1.43	0.51
**2**	Maleic acid	*	*	1.93	0.95
**3**	D-Cysteine	**	0.06	2.09	1.06
**4**	N-Acetylasparagine	*	*	2.58	1.37
**5**	3-Hydroxy-2-methylpyridine-4,5-dicarboxylate	**	*	1.86	0.89
**6**	Dihydroneopterin phosphate	*	*	1.65	0.73

[1] The fold change analysis was conducted based on the SMFs of ovarian cancer patients before and after chemotherapy.

References

1. Wang, X.; Yao, W.; Guo, R.; Yang, X.; Tang, J.; Zhang, J.; Gao, W.; Timchenko, V.; Liu, J., Soft and Moldable Mg-Doped Liquid Metal for Conformable Skin Tumor Photothermal Therapy. *Adv. Healthc. Mater.* **2018,** *7* (14), 1800318.
2. Chen, X.; Zhang, Q.; Li, J.; Yang, M.; Zhao, N.; Xu, F.-J., Rattle-Structured Rough Nanocapsules with in-Situ-Formed reil Gold Nanorod Cores for Complementary Gene/Chemo/Photothermal Therapy. *ACS Nano* **2018,** *12* (6), 5646-5656.
3. Song, J.; Yang, X.; Yang, Z.; Lin, L.; Liu, Y.; Zhou, Z.; Shen, Z.; Yu, G.; Dai, Y.; Jacobson, O.; Munasinghe, J.; Yung, B.; Teng, G.-J.; Chen, X., Rational Design of Branched Nanoporous Gold Nanoshells with Enhanced Physico-Optical Properties for Optical Imaging and Cancer Therapy. *ACS Nano* **2017,** *11* (6), 6102-6113.
4. Niu, Y.; Yu, M.; Hartono, S. B.; Yang, J.; Xu, H.; Zhang, H.; Zhang, J.; Zou, J.; Dexter, A.; Gu, W.; Yu, C., Nanoparticles Mimicking Viral Surface Topography for Enhanced Cellular Delivery. *Adv. Mater.* **2013,** *25* (43), 6233-6237.
5. Kocer, G.; ter Schiphorst, J.; Hendrikx, M.; Kassa, H. G.; Leclere, P.; Schenning, A. P. H. J.; Jonkheijm, P., Light-Responsive Hierarchically Structured Liquid Crystal Polymer Networks for Harnessing Cell Adhesion and Migration. *Adv. Mater.* **2017,** *29* (27), 1606407.
6. Zhou, Q.; Xie, J.; Bao, M.; Yuan, H.; Ye, Z.; Lou, X.; Zhang, Y., Engineering aligned electrospun PLLA microfibers with nano-porous surface nanotopography for modulating the responses of vascular smooth muscle cells. *J. Mat. Chem. B* **2015,** *3* (21), 4439-4450.
7. Li, S.; Zhao, S.; Pei, J.; Wang, H.; Meng, H.; Vrouwenvelder, J. S.; Li, Z., Stimuli-Responsive Lysozyme Nanocapsule Engineered Microfiltration Membranes with a Dual-Function of Anti-Adhesion and Antibacteria for Biofouling Mitigation. *ACS Appl. Mater. Interfaces* **2021,** *13* (27), 32205-32216.
8. Xu, Y.; Wen, W.; Wu, J.-M., Titania nanowires functionalized polyester fabrics with enhanced photocatalytic and antibacterial performances. *J. Hazard. Mater.* **2018,** *343*, 285-297.
9. Nguyen, D. H. K.; Loebbe, C.; Linklater, D. P.; Xu, X.; Vrancken, N.; Katkus, T.; Juodkazis, S.; Maclaughlin, S.; Baulin, V.; Crawford, R. J.; Ivanova, E. P., The idiosyncratic self-cleaning cycle of bacteria on regularly arrayed mechano-bactericidal nanostructures. *Nanoscale* **2019,** *11* (35), 16455-16462.
10. Shi, J.; Wang, L.; Dai, Z.; Zhao, L.; Du, M.; Li, H.; Fang, Y., Multiscale Hierarchical Design of a Flexible Piezoresistive Pressure Sensor with High Sensitivity and Wide Linearity Range. *Small* **2018,** *14* (27).
11. Qi, Y.; Cao, Y.; Meng, X.; Cao, J.; Li, X.; Hao, Q.; Lei, W.; Li, Q.; Li, J.; Si, W., Facile synthesis of 3D sulfur/nitrogen co-doped graphene derived from graphene oxide hydrogel and the simultaneous determination of hydroquinone and catechol. *Sens. Actuators B Chem.* **2019,** *279*, 170-176.
12. Shu, W.; Wang, Y.; Liu, C.; Li, R.; Pei, C.; Lou, W.; Lin, S.; Di, W.; Wan, J., Construction of a Plasmonic Chip for Metabolic Analysis in Cervical Cancer Screening and Evaluation. *Small Methods* **2020,** *4* (4), 1900469.
13. Tseng, Y.-T.; Chang, H.-Y.; Harroun, S. G.; Wu, C.-W.; Wei, S.-C.; Yuan, Z.; Chou, H.-L.; Chen, C.-H.; Huang, C.-C.; Chang, H.-T., Self-Assembled Chiral Gold Supramolecules with Efficient Laser Absorption for Enantiospecific Recognition of Carnitine. *Anal. Chem.* **2018,** *90* (12), 7283-7291.
14. Yoon, S.; Park, S.; Kim, M. S.; Lee, C. Y., Concomitant desalting and concentration of neuropeptides on a donut-shaped surface pattern for MALDI mass spectrometry. *ChemComm*

2018, *54* (45), 5688-5691.
15. Li, W.; Khan, M.; Li, H.; Lin, L.; Mao, S.; Lin, J.-M., Homogenous deposition of matrix-analyte cocrystals on gold-nanobowl arrays for improving MALDI-MS signal reproducibility. *ChemComm* **2019,** *55* (15), 2166-2169.
16. Ge, K.; Peng, Y.; Lu, Z.; Hu, Y.; Li, G., Aptamer-gold nanoparticle doped covalent organic framework followed by matrix-assisted laser desorption/ionization time-of-flight mass spectrometry for selective enrichment and detection of human insulin. *J. Chromatogr. A* **2020,** *1615*.
17. Huang, S.; Chen, G.; Ou, R.; Qin, S.; Wang, F.; Zhu, F.; Ouyang, G., Ultrathin Self-Assembled Diphenylalanine Nanosheets through a Gold-Stabilized Strategy for High-Efficiency Adsorption/Desorption/Ionization. *Anal. Chem.* **2018,** *90* (14), 8607-8615.
18. Wang, X.-N.; Tang, W.; Gordon, A.; Wang, H.-Y.; Xu, L.; Li, P.; Li, B., Porous TiO2 Film Immobilized with Gold Nanoparticles for Dual-Polarity SALDI MS Detection and Imaging. *ACS Appl. Mater. Interfaces* **2020,** *12* (38), 42567-42575.
19. Kim, M.-J.; Yun, T. G.; Noh, J.-Y.; Kang, M.-J.; Pyun, J.-C., Photothermal Structural Dynamics of Au Nanofurnace for In Situ Enhancement in Desorption and Ionization. *Small* **2021,** *17* (49), 2103745.
20. Kim, M.-J.; Yun, T. G.; Noh, J.-Y.; Song, Z.; Kim, H.-R.; Kang, M.-J.; Pyun, J.-C., Laser-Induced Surface Reconstruction of Nanoporous Au-Modified TiO2 Nanowires for In Situ Performance Enhancement in Desorption and Ionization Mass Spectrometry. *Adv. Funct. Mater.* **2021,** *31* (29), 2102475.
21. Kim, M.-J.; Yun, T. G.; Noh, J.-Y.; Park, J.-M.; Kang, M.-J.; Pyun, J.-C., Synergistic Effect of the Heterostructure of Au Nanoislands on TiO2 Nanowires for Efficient Ionization in Laser Desorption/Ionization Mass Spectrometry. *ACS Appl. Mater. Interfaces* **2019,** *11* (22), 20509-20520.
22. Li, L.; Qiu, Z.; Qi, Y.; Zhao, D.; Ali, I.; Sun, C.; Xu, L.; Zheng, Z.; Ma, C., AuNPs/NiFe-LDHs-assisted laser desorption/ionization mass spectrometry for efficient analysis of metronidazole and its metabolites in water samples. *J. Hazard. Mater.* **2022,** *423*, 126893.
23. Alexandra Iakab, S.; Rafols, P.; Tajes, M.; Correig-Blanchar, X.; Garcia-Altares, M., Gold Nanoparticle-Assisted Black Silicon Substrates for Mass Spectrometry Imaging Applications. *ACS Nano* **2020,** *14* (6), 6785-6794.
